# Macrophage-derived KIF13B interacts with USP9X to attenuate abdominal aortic aneurysm development by potentiating TFEB stability

**DOI:** 10.7150/thno.118958

**Published:** 2025-08-16

**Authors:** Jingxuan Chen, Yitong Xu, Huahui Yu, Yiran Liu, Guolin Miao, Yufei Han, Liwen Zheng, Zeyu Cai, Zihao Zhou, Jinxuan Chen, Sijing Shi, Pingping Lai, Wenxi Zhang, Lianxin Zhang, Si Mei, Yinqi Zhao, Ling Zhang, Wei Huang, Yuhui Wang, Dongyu Zhao, Wei Kong, Yanwen Qin, Erdan Dong, Xunde Xian

**Affiliations:** 1 Institute of Cardiovascular Sciences, State Key Laboratory of Vascular Homeostasis and Remodeling, School of Basic Medical Sciences, Peking University, Beijing, China.; 2 The Key Laboratory of Remodeling-Related Cardiovascular Diseases, Ministry of Education, Beijing Anzhen Hospital, Capital Medical University, Beijing Institute of Heart Lung and Blood Vessel Disease, Beijing, China.; 3 Department of Biomedical Informatics, School of Basic Medical Sciences, State Key Laboratory of Vascular Homeostasis and Remodeling, Peking University, Beijing, China.; 4 Department of Cardiology and Institute of Vascular Medicine, Peking University Third Hospital, Beijing, China.; 5 Department of Physiology and Pathophysiology, School of Basic Medical Sciences, State Key Laboratory of Vascular Homeostasis and Remodeling, Peking University, Beijing, China.; 6 Research Center for Cardiopulmonary Rehabilitation, University of Health and Rehabilitation Sciences Qingdao Hospital (Qingdao Municipal Hospital), School of Health and Life Sciences, University of Health and Rehabilitation Sciences, 369 Dengyun Road, Qingdao, China.; 7 Beijing Key Laboratory of Cardiovascular Receptors Research, Peking University Third Hospital, Beijing, China.

**Keywords:** abdominal aortic aneurysm, KIF13B, macrophage, senescence, lysosome

## Abstract

**Rationale:** Abdominal aortic aneurysm (AAA) is a highly lethal cardiovascular disorder for which there is no effective medication to date. Kinesin family member 13b (KIF13B), a vital motor protein, has been recently identified as a novel regulator of lipid metabolism. However, the role of KIF13B in AAA development has not been documented.

**Methods:** We determined the expression of KIF13B in aortic tissues from clinical patients and porcine pancreatic elastase (PPE) or angiotensin II (ANG II)-induced AAA mouse models. To investigate the influence of KIF13B on AAA expansion, we established global, myeloid cell-specific and vascular smooth muscle cell (VSMC)-specific conditional *Kif13b*-deficient mice in PPE and/or ANG II-induced AAA models.

**Results:** RNA-seq data from GEO database (GSE57691) revealed a significant decrease in *KIF13B* gene expression within the aortic tissues of patients with AAA. KIF13B protein levels were largely reduced in aortic tissue samples from patients and two mouse models with AAA. Complete inactivation of *Kif13b* or depleting *Kif13b* from myeloid cells but not smooth muscle cells (SMCs) exacerbated AAA development. Mechanistic studies identified transcription factor EB (TFEB) as a critical downstream target of KIF13B. KIF13B stabilized and upregulated TFEB by enhancing its deubiquitination through an interaction with deubiquitinase USP9X to maintain the proper function of lysosomes, thus inhibiting the senescence-associated secretory phenotype (SASP) and proinflammatory response of macrophages. Moreover, restoration of macrophage *Kif13b* or senolytic therapy dramatically mitigated AAA expansion *in vivo*.

**Conclusions:** In the present study, we provided a new insight into the pathogenesis of AAA and defined a KIF13B-USP9X-TFEB axis that is essential for the regulation of macrophage function, suggesting that macrophage-derived *Kif13b* is a beneficial regulator of vascular homeostasis and targeting KIF13B could be a potential therapeutic approach for the treatment of human AAA disease in future clinical trial.

## Introduction

Abdominal aortic aneurysm (AAA) is a fatal vascular disease characterized by a localized, permanent dilatation of the abdominal aorta exceeding 1.5 times its normal diameter 1. Major risk factors include hypertension, hypercholesterolemia, atherosclerosis, smoking, advanced age, and male sex 2. Notably, AAA rupture carries a mortality rate exceeding 80% 1; yet, no FDA-approved pharmacological therapies exist to slow AAA progression or reduce rupture risk. Current management relies on surgical repair or endovascular stenting, which is viable for fewer than 10% of patients 3, leaving the majority at significant risk. Thus, elucidating novel pathogenic mechanisms and identifying therapeutic targets for AAA remain urgent priorities.

The pathophysiology of AAA involves inflammatory cell infiltration into the vascular wall, vascular smooth muscle cell (VSMC) apoptosis, and degradation of extracellular matrix elastin 4. Growing evidence implicates dysregulated lipid metabolism, particularly involving triglyceride-rich lipoproteins (TRLs), in AAA pathogenesis 4-8. Genome-wide association studies have identified 121 AAA risk loci, including regulators of lipoprotein receptors such as *PCSK9* 9. Moreover, both clinical and preclinical studies further underscore the role of lipoprotein receptors (e.g., low-density lipoprotein receptor-associated protein 1, LRP1) in AAA development 10-14, highlighting the critical contribution of dysfunctional lipid metabolism to this devastating disease.

The kinesin-3 family, known for its role in intracellular transport of organelles and macromolecules 15, includes kinesin family member 13b (KIF13B), the largest family member. Emerging evidence demonstrates that KIF13B serves as a novel modulator of lipoprotein receptor function, specifically localized to lipid rafts in hepatocyte membranes where it colocalizes with LRP1 to regulate lipid metabolism 16. While KIF13B with a high expression level in endothelial cells and metabolic organs has been reported to regulate vascular permeability and angiogenesis 17-20, its precise function in vascular homeostasis remains poorly understood. Moreover, our prior work has identified KIF13B as a novel modulator of lipid metabolism, essential for hepatic lipid homeostasis and protection against metabolic dysfunction-associated fatty liver disease (MAFLD) 21. Given the established links between dyslipidemia, MAFLD, and cardiovascular disease (CVD) 22,23, we herein hypothesized that KIF13B might influence AAA pathogenesis. However, the fundamental role of KIF13B in AAA and its potential as a therapeutic target need to be further explored.

In this study, we investigate the involvement of KIF13B in AAA development and its underlying molecular mechanisms. Our key findings demonstrate that macrophage-specific KIF13B stabilizes transcription factor EB (TFEB), a master regulator of lysosomal biogenesis, via enhancing the deubiquitylation mediated by ubiquitin specific peptidase 9 x-linked (USP9X), to attenuate pro-inflammatory polarization and senescence of macrophages, ultimately suppressing AAA progression.

## Methods

### Human studies

All procedures involving human participants adhered to the principles of the Declaration of Helsinki and were approved by the Medical Ethics Committee of Beijing Anzhen Hospital (Beijing, China). Written informed consent was obtained from all patients prior to sample collection. Aortic tissue samples were collected from six AAA patients undergoing open surgical repair, with adjacent non-aneurysmal aortic tissue from the same patients serving as controls. Tissues were fixed in 10% neutral-buffered formalin, paraffin-embedded, and sectioned at 5 µm thickness 24.

### Animal studies

All animal experiments complied with the National Institutes of Health Guide for the Care and Use of Laboratory Animals (8th edition, 2011) and were approved by the Animal Ethics Committee of Peking University (Protocol LA2023460). Global *Kif13b* knockout (*Kif13b*^-/-^) mice and floxed *Kif13b* (*Kif13b*^f/f^) mice were generated using CRISPR/Cas9 technology by the Institute of Laboratory Animal Science, Chinese Academy of Medical Sciences. Conditional myeloid cell-specific *Kif13b* knockout (*Kif13b*^f/f^*Lyz2-Cre*, *Lyz2Kif13b*^f/f^) mice and VSMC-specific *Kif13b* knockout (*Kif13b*^f/f^*Sm22-Cre*, *Sm22Kif13b*^f/f^) mice were generated by breeding *Kif13b*^f/f^ mice with *Lyz2-Cre* and *Sm22-Cre* mice provided as a gift from Prof. Hongliang Li (Wuhan University), respectively. All mice were housed under specific pathogen-free conditions at the Peking University Health Science Center Animal Facility.

### Statistical analysis

All statistical analysis was performed using GraphPad Prism version 9.0 (GraphPad Software). Continuous data are presented as mean ± SEM. Survival of AAA mice was examined using the Kaplan-Meier survival curve and differences were analyzed using the log-rank test (Mantel-Cox). Normality of data distribution was assessed by the Shapiro-Wilk test and equality of variance by the Brown-Forsythe test. For comparisons between two groups, differences were compared using unpaired Student's t-test (normal distribution and equal variance) or Mann-Whitney U-test (non-normal distribution) or Welch t-test (unequal variances). For multiple comparisons of equal variance (> 2 groups), one-way ANOVA analyses (single variable) or two-way ANOVA analyses (multiple variables) were used, followed by Tukey post hoc tests (equal variance). Kruskal-Wallis test and Bonferroni post hoc test were used for multiple tests of unequal variance. Pearson correlation analysis was used to assess the correlation between the expression levels of the 2 genes, values of P < 0.05 were considered statistically significant.

Detailed methodologies are provided in the Supplemental Material.

## Results

### KIF13B expression is decreased in aortic tissues of AAA patients and mouse models

**Figure 1:**
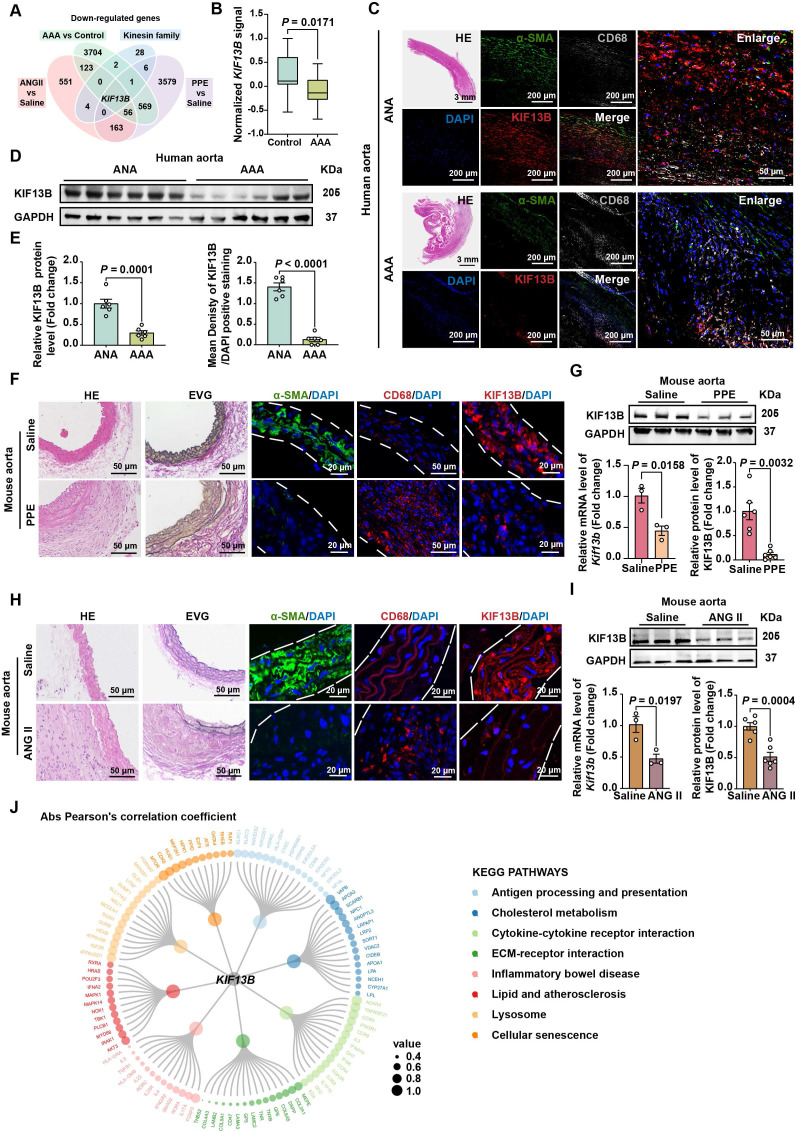
** KIF13B expression is decreased in aortic tissues of AAA patients and mouse models. (A)** The Venn diagram illustrates the intersection of commonly downregulated genes and kinesin family member genes across different AAA samples and control tissues, including: the murine ANG II-AAA model (GSE265897), murine PPE-AAA model (GSE51228), and AAA patients (GSE57691). Genes with *FDR* < 0.05 and log_2_foldchange < 0 were included. **(B)** Normalized *KIF13B* signal in aortic samples comparing healthy controls (HC, n = 9) and abdominal aortic aneurysm (AAA, n = 44) patients within the GEO dataset (GSE57691). **(C)** Representative images of HE staining and co-localized immunofluorescence staining depict α-SMA (green), CD68 (grey), and KIF13B (red) protein levels in human AAA lesions versus adjacent normal aortas (ANAs) (*n* = 6/group). **(D)** Western blot analysis of KIF13B protein levels in human AAA lesions and ANAs (*n* = 6/group). **(E)** Quantitative analysis of KIF13B protein expression levels corresponding to **C** and **D**. **(F)** Representative images of HE staining, EVG staining, and immunofluorescence staining depict α-SMA (green), CD68 (red), and KIF13B (red) expression levels in WT mouse abdominal aortas treated with saline (*n* = 3) or porcine pancreatic elastase (PPE, *n* = 6). **(G)** qPCR analysis of *Kif13b* mRNA expression (*n* = 3/group) and western blot analysis of KIF13B protein expression in WT mouse abdominal aortas treated with saline or PPE (*n* = 6/group). **(H)** Representative images of HE staining, EVG staining, and immunofluorescence staining illustrate α-SMA (green), CD68 (red), and KIF13B (red) expression levels in WT mouse abdominal aortas perfused with saline (*n* = 3) or angiotensin II (ANG II, *n* = 6). **(I)** qPCR analysis of *Kif13b* mRNA expression (*n* = 3/group) and western blot analysis of KIF13B protein expression in WT mouse abdominal aortas perfused with saline or ANG II (*n* = 3/group). **(J)** Correlation analysis of TMM-normalized counts between *KIF13B* and other genes in KEGG pathways. Node sizes represent the absolute values of Pearson's correlation coefficients. Data are presented as mean ± SEM. **B**, **E**, **G left**, **i** were analyzed by unpaired Student's *t*-test; **G right** was analyzed by Welch's *t*-test.

To delineate the role of kinesin family member genes in AAA pathogenesis, we systematically performed differentially expressed genes (DEGs) analysis across multiple AAA models. Cross-model comparative analysis revealed that *KIF13B* gene was the sole kinesin family member exhibiting consistent and significant downregulation across all AAA variants (Figure 1A). To confirm the association between KIF13B and AAA, we analyzed bulk RNA-seq data from the GEO database (GSE57691), comprising aortic tissues from 44 AAA patients and 9 healthy controls. *KIF13B* mRNA levels were significantly downregulated in AAA tissues compared to controls (Figure 1B). Immunofluorescence staining and Western blot analysis corroborated these findings, showing reduced KIF13B protein expression in human AAA lesions, in which the positive staining content of α-SMA was markedly reduced in the AAA lesions, representing a loss of VSMC, whereas more CD68-positive macrophages were accumulated, indicating an enhanced inflammatory status, compared to adjacent normal aortas (ANAs) (Figure 1C-E). In addition, when compared to healthy control tissues, we also observed a significant decrease in KIF13B expression at both mRNA and protein levels in PPE-induced AAA mouse model as well as in the ANG II-induced AAA murine model in the context of hyperlipidemia by delivering adeno-associated virus 8 (AAV8) expressing PCSK9^D337Y^ through tail vein, followed by a Western diet (WD) challenge (Figure 1F-I). Furthermore, to explore the functional relevance of KIF13B in AAA, we performed gene set enrichment analysis (GSEA) using the AAA-associated transcriptome. *KIF13B* expression strongly correlated with pathways linked to inflammation, lysosomal function, and cellular senescence, all established contributors to AAA pathogenesis 25 (Figure 1J). Taken together, these findings suggest a negative correlation between KIF13B expression levels and the incidence of AAA development.

### Global inactivation of *Kif13b* aggravates AAA development *in vivo*

**Figure 2:**
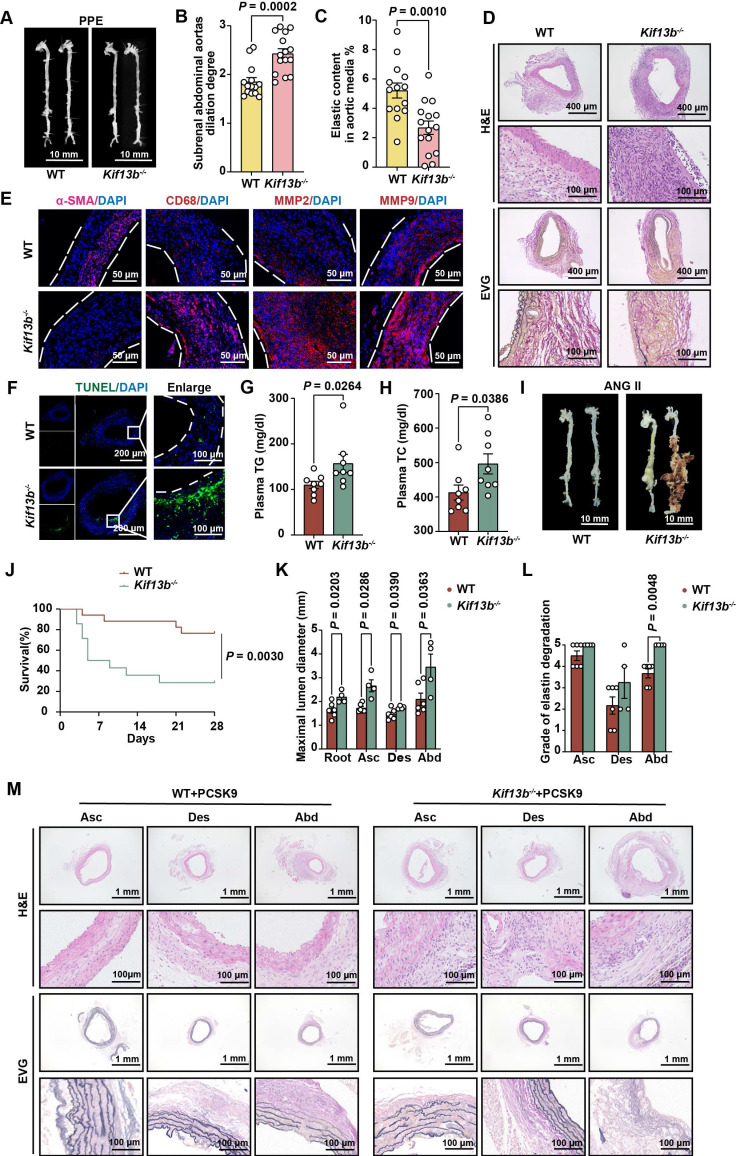
** Global inactivation of *Kif13b* aggravates AAA development *in vivo*. (A-F)** PPE-Induced AAA Model: Eight-week-old male WT (*n* = 14) and *Kif13b*^-/-^ (*n* = 15) mice were subjected to PPE-induced AAA. **(A)** Representative macroscopic images of AAA in each group. **(B)** Quantitative analysis of the maximal AAA diameter normalized to untreated aorta (Mann-Whitney *U*-test). **(C)** Residual elastic fiber area relative to vessel wall area (unpaired Student's *t*-test). **(D)** Representative HE and EVG staining of aortic sections. **(E)** Immunofluorescence staining of α-SMA (pink), CD68 (red), MMP2 (red), and MMP9 (red).** (F)** TUNEL staining (green) for apoptosis detection. **(G-M)** ANG II-Induced AAA Model with Hyperlipidemia: Eight-week-old male WT (*n* = 17) and *Kif13b*^-/-^ (*n* = 14) mice underwent ANG II infusion combined with AAV8-PCSK9 injection and Western diet. **(G-H)** Plasma triglyceride (TG) and total cholesterol (TC) levels (*n* = 8/group; Mann-Whitney *U*-test for TG, unpaired *t*-test for TC). **(I)** Macroscopic AAA features. **(J)** Survival analysis (Kaplan-Meier method, log-rank test).** (K)** Maximal aortic diameter across segments (ascending [Asc], descending [Des], abdominal [Abd]; Welch's *t*-test for Asc, unpaired *t*-test for Des/Abd). **(L)** Elastin degradation severity (Mann-Whitney *U*-test). **(M)** Representative HE and EVG staining. Abbreviations: Asc, ascending aorta; Des, descending aorta; Abd, abdominal aorta. Data are mean ± SEM. Statistical methods are specified per panel.

To determine whether reduced KIF13B expression drives AAA pathogenesis or represents a compensatory response, we generated global *Kif13b* knockout (*Kif13b*^-/-^) mice. Following PPE induction, *Kif13b*^-/-^ mice exhibited modestly elevated plasma cholesterol levels (Table S1) and severely aggravated AAA phenotypes showing the increased maximal diameter of the infrarenal abdominal aorta with mean value of 2.43 mm compared to wild-type (WT) mice with mean value of 1.85 mm (Figure 2A-B). Histological staining revealed an increase in vessel wall thickness and elastic fiber degradation in *Kif13b*^-/-^ mice (Figure 2C-D), accompanied by reduced a-SMA-positive areas and more CD68 staining (Figure 2E). Since matrix metalloproteinase 2 (MMP2) and matrix metalloproteinase 9 (MMP9) are two key enzymes responsible for elastin destruction, we also examined the protein levels of MMP2 and MMP9 by immunofluorescence. Our result showed that loss of KIF13B led to the elevated levels of MMP2 and MMP9 (Figure 2E). Moreover, using terminal deoxynucleotidyl transferase dUTP nick-end labelling (TUNEL) staining, we found that depleting *Kif13b* promoted apoptosis in the aortic walls (Figure 2F).

To further confirm the deleterious effect of *Kif13b* deletion on AAA in a hyperlipidemic context, we also conducted a similar process to induce AAA mouse model using ANG II combined with AAV8-PCSK9^D337Y^ injection and Western diet treatment in WT and *Kif13b*^-/-^ mice. We found that *Kif13b*^-/-^ mice exhibited markedly elevated plasma levels of triglyceride (TG) and total cholesterol (TC) (Figure 2G-H). ANG II infusion increased systolic and diastolic blood pressure to the same extent in WT and *Kif13b*^-/-^ mice (Table S2). Survival curve analysis showed that the mortality rate of *Kif13b*^-/-^ mice infused by ANG II was as high as 71.4%, which was three times higher than that of WT mice with mortality rate of 23.5 (Figure 2J). Next, we performed segmental analysis of aortic diameters in surviving mice and confirmed that *Kif13b* deletion resulted in significant enlargement of aortic root, ascending and abdominal aortic segments (Figure 2I-K) as well as increased vessel wall thickness and more disrupted elastic fibers (Figure 2L-M). These observations demonstrate that global *Kif13b* deletion promotes the development of AAA *in vivo*.

### KIF13B deficiency exacerbates the enrichment of pro-inflammatory macrophages in AAA

**Figure 3:**
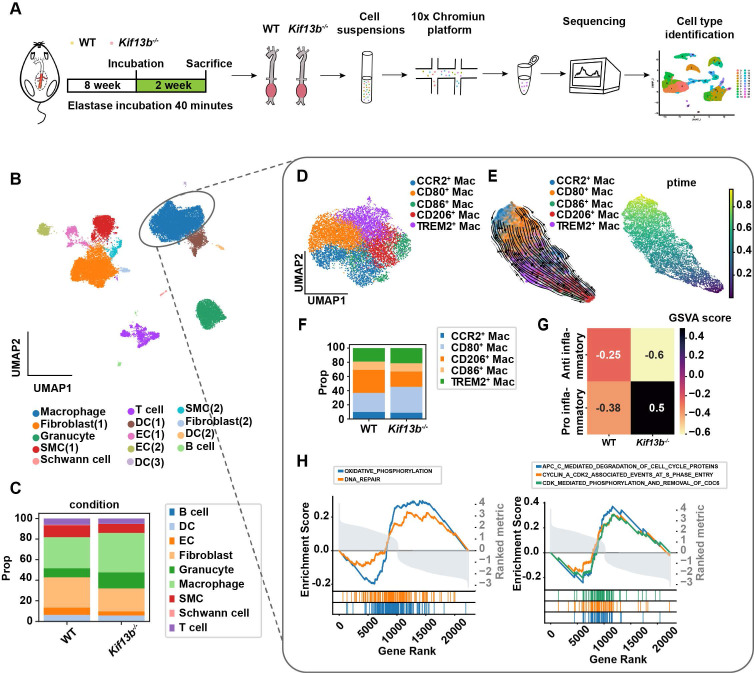
** KIF13B deficiency exacerbates the enrichment of pro-inflammatory macrophages in AAA. (A)** Schematic of the single-cell sequencing experimental design. Aortic tissues from PPE-treated WT (*n* = 10) and *Kif13b*^-/-^ (*n* = 9) mice were pooled into a single sample for sequencing. **(B)** Uniform Manifold Approximation and Projection (UMAP) plot depicting nine distinct cell clusters in AAA tissues, color-coded by cell type. **(C)** Proportional distribution of all cell types in each experimental group. **(D)** UMAP plot highlighting five macrophage subpopulations, color-labeled for differentiation. **(E)** Pseudotime analysis illustrating the differentiation trajectories of macrophage clusters shown in** D**. **(F)** Proportional distribution of macrophage subpopulations in each group.** (G)** Gene Set Variation Analysis (GSVA) of pro-inflammatory and anti-inflammatory gene signatures in each group. **(H)** Gene Set Enrichment Analysis (GSEA) of differentially expressed genes (DEGs) from **D**, using Hallmark and Reactome pathway databases. Pathways with a normalized enrichment score (NES) > 1.5 and *P* < 0.05 were considered significantly enriched.

Of note that multiple cell types are involved in AAA formation. To delineate the cellular mechanisms underlying AAA exacerbation in *Kif13b* deficiency, we performed a single-cell sequencing (scRNA-seq) analysis on AAA tissues from 8-week-old male WT and *Kif13b*^-/-^ mice subjected to PPE-induced AAA (Figure 3A). Unsupervised clustering of conserved gene expression profiles identified nine distinct cell populations, including macrophages, fibroblasts, smooth muscle cells (SMCs), and endothelial cells (Figure 3B), which were confirmed using canonical markers (Figure S1A). Strikingly, macrophages accounted for 50% of all cells in the AAA tissue of *Kif13b*^-/-^ mice, a significant increase compared to WT controls (Figure 3C and Figure S1B), indicating that *Kif13b* deletion amplifies macrophage enrichment in AAA lesions.

scRNA-seq analysis further stratified macrophages into five distinct subpopulations, including three pro-inflammatory M1-like types marked by elevated CCR2, CD80, and CD86 and two anti-inflammatory M2-like types expressing CD206 and TREM2 (Figure 3D). Pseudotime trajectory analysis revealed a phenotypic shift from M2-like to M1-like macrophages during AAA progression (Figure 3E). Strikingly, *Kif13b* deletion skewed this balance, increasing the proportion of CD80-high pro-inflammatory macrophages while reducing CD206-high anti-inflammatory populations (Figure 3F, Figure S1C-E). Moreover, Gene Set Variation Analysis (GSVA) confirmed a pro-inflammatory state in *Kif13b*-deficient macrophages (Figure 3G). Consistently, complementary Gene Set Enrichment Analysis (GSEA) linked *Kif13b* deficiency to activation of oxidative phosphorylation, DNA repair, and cell cycle pathways (Figure 3H), suggesting KIF13B governs macrophage metabolic reprogramming and proliferative capacity to modulate polarization.

### Loss of myeloid cell-specific KIF13B promotes AAA formation

**Figure 4:**
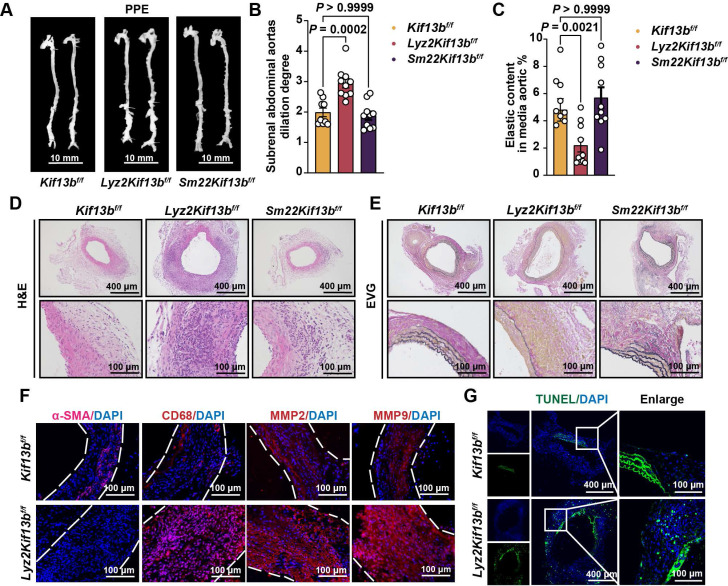
** Loss of myeloid cell-specific KIF13B promotes AAA formation. (A-E)** Eight-week-old male *Kif13b*^f/f^ (n = 9), *Lyz2*^Cre^*Kif13b*^f/f^ (n = 10), and *Sm22α*^Cre^*Kif13b*^f/f^ (n = 10) mice were subjected to PPE-induced AAA. **(A)** Representative macroscopic images of AAA lesions. **(B)** Maximal AAA diameter normalized to untreated aorta. **(C)** Residual elastic fiber area relative to vessel wall area. **(D-E)** Representative HE and EVG staining of aortic sections. **(F-G)** Additional analyses in *Kif13b*^f/f^ and *Lyz2*^Cre^*Kif13b*^f/f^ mice. **(F)** Immunofluorescence staining of α-SMA (pink), CD68 (red), MMP2 (red), and MMP9 (red). **(G)** TUNEL staining (green) for apoptosis detection. Data are presented as mean ± SEM and analyzed by one-way ANOVA with Bonferroni post-hoc tests.

To validate scRNA-seq findings, we constructed loxp flanked *Kif13b* (*Kif13b*^f/f^) mice, which were bred with the myeloid or smooth muscle cell-specific *Cre* transgenic mice to obtain macrophage (*Lyz2Kif13b*^f/f^) and smooth muscle cell (*Sm22Kif13b*^f/f^) specific-*Kif13b* conditional knockout mice, respectively. Both lines were subjected to PPE-induced AAA to evaluate the role of KIF13B in AAA development. Plasma lipid profiles remained unchanged across genotypes (Table S 1). As expected, mice lacking macrophage-derived *Kif13b* showed exacerbated AAA progression with increased aortic dilation, elastin degradation, and inflammatory infiltration; however, in contrast, depleting *Kif13b* from smooth muscle cells had no significant influence on AAA development (Figure 4A-G).

### KIF13B maintains macrophage homeostasis by suppressing senescence

To investigate the relationship between KIF13B and macrophage inflammation, we analyzed LPS-induced pro-inflammatory polarization *in vitro*. LPS treatment significantly reduced KIF13B mRNA and protein levels in differentiated human acute monocytic leukemia cells (THP1 macrophages) (Figure S2A-C). Similarly, KIF13B expression was also significantly decreased in LPS-induced primary mouse bone marrow-derived macrophages (BMDMs) at both the mRNA and protein levels (Figure S2D-F).

**Figure 5:**
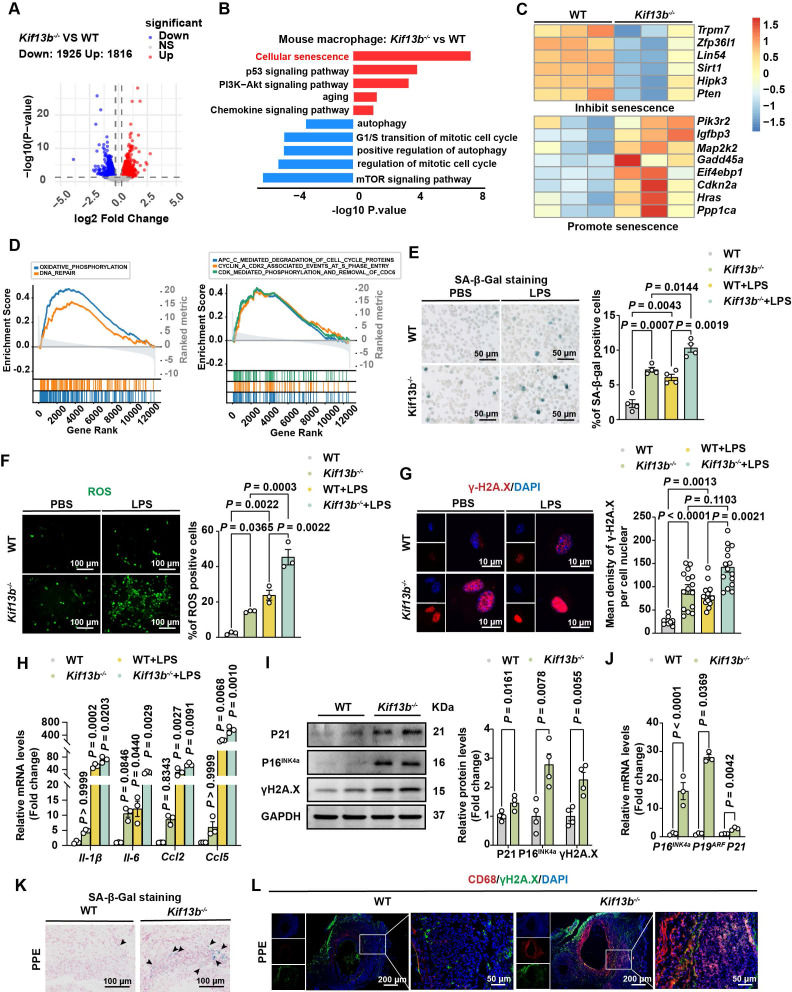
** KIF13B maintains macrophage homeostasis by suppressing senescence. (A)** Volcano plot displaying differentially expressed genes (DEGs) in bone marrow-derived macrophages (BMDMs) from WT and *Kif13b*^-/-^ mice (n = 3/group). Genes meeting significance thresholds (*P* < 0.05, |Fold change| > 1.2) are highlighted (1,925 upregulated in red; 1,816 downregulated in blue). **(B)** Bar plot showing significantly enriched KEGG and Gene Ontology (GO) pathways among DEGs. **(C)** Heatmap of senescence-related DEGs (pro- and anti-senescence genes) between genotypes. **(D)** Gene Set Enrichment Analysis (GSEA) of hallmark and Reactome pathways (significant enrichment: NES > 1.5, *P* < 0.05). **(E-J)** LPS-stimulated (10 ng/ml, 24h) BMDMs from 8-week-old male WT and *Kif13b*^-/-^ mice. **(E)** SA-β-gal staining (representative images and quantification, n = 4). **(F)** ROS detection (fluorescence images and quantification, 3 independent experiments). **(G)** γ-H2A.X immunofluorescence (images and nuclear density quantification, n = 3). **(H)** qPCR analysis of SASP-related genes (n = 3). **(I)** Western blot of senescence markers (P21, P16, γ-H2A.X; n = 4). **(J)** qPCR analysis of cell cycle regulators (n = 3).** (K-L)** *In vivo* analysis of PPE-induced AAA. **(K)** SA-β-gal staining in aortic tissues (n = 6). **(L)** Immunofluorescence of CD68^+^ macrophages with γ-H2A.X (DNA damage marker). Data presented as mean ± SEM. **E-G, H** were analyzed by two-way ANOVA with Bonferroni post-hoc tests. **I-J P19^ARF^ and P21** were analyzed by unpaired Student's t-test. **I-J P16^INK4a^** was analyzed by Welch's *t*-test.

We next defined the molecular mechanisms linking *Kif13b* deficiency to pro-inflammatory macrophage polarization by performing bulk RNA-seq on BMDMs from WT and *Kif13b^-/-^* mice (Figure 5A). KEGG and Gene Ontology (GO) analyses revealed pronounced activation of cellular senescence pathways in *Kif13b*^-/-^ macrophages with an upregulation of pro-senescent genes and a downregulation of anti-aging genes (Figure 5B-C). Consistent with *in vivo* findings, *Kif13b*^-/-^ BMDMs exhibited enhanced oxidative phosphorylation (OXPHOS), DNA repair, and cell cycle dysregulation (Figure 5D). Collectively, these data identify cellular senescence as a pivotal mechanism by which *Kif13b* deficiency promotes macrophage inflammation, then driving AAA progression.

Given the established link between aging and AAA risk, we assessed whether *Kif13b* deletion promoted macrophage senescence using senescence-associated β-galactosidase (SA-β-gal) activity 26. The result from SA-β-gal staining demonstrated that macrophage senescence was significantly driven by silencing *Kif13b* with or without LPS treatment (Figure 5E). In addition, ROS levels were elevated in *Kif13b^-/-^* macrophages under both basal and LPS-treated conditions (Figure 5F). Consistently, more γ-H2A. X, another hallmark of senescent cells, was prominently aggregated in the nucleus (Figure 5G). LPS-stimulated macrophages with *Kif13b* deficiency displayed worsened severity of senescence-associated secretory phenotype (SASP) with increased mRNA expression of *Il-1β* and *Il-6*, which can be used as a biomarker of senescence. The severity of SASP was worsened in *Kif13b*-deficient macrophages stimulated with LPS (Figure 5H), and upregulated expression of cell cycle regulators, including Cdkn1a (P21) and Cdkn2a (P16^INK4a^ and P19^ARF^), at both mRNA and protein levels (Figure 5I-J). In accordance, SA-β-gal activity was elevated in aneurysmal tissue of *Kif13b^-/-^* mice deletion *in vivo* (Figure 5K). Immunofluorescence results further demonstrated that loss of *Kif13b* exacerbated macrophage senescence as evidenced by co-localization of CD68 and γ-H2A.X (Figure 5L).

Considering the therapeutic potential of senolytics in cardiovascular diseases, we tested quercetin, a well-established senolytic agent 27, in *Kif13b*^-/-^ BMDMs. Our results revealed that quercetin effectively reduced the number of SA-β-gal positive cells in a dose-dependent manner and concurrently downregulated the expression of pro-inflammatory genes (Figure S3A-B). These findings confirm that *Kif13b* deficiency drives macrophage senescence and pro-inflammatory polarization and demonstrate that senolytic intervention mitigates these effects.

### KIF13B maintains lysosomal function to inhibit macrophage senescence *in vitro*

**Figure 6:**
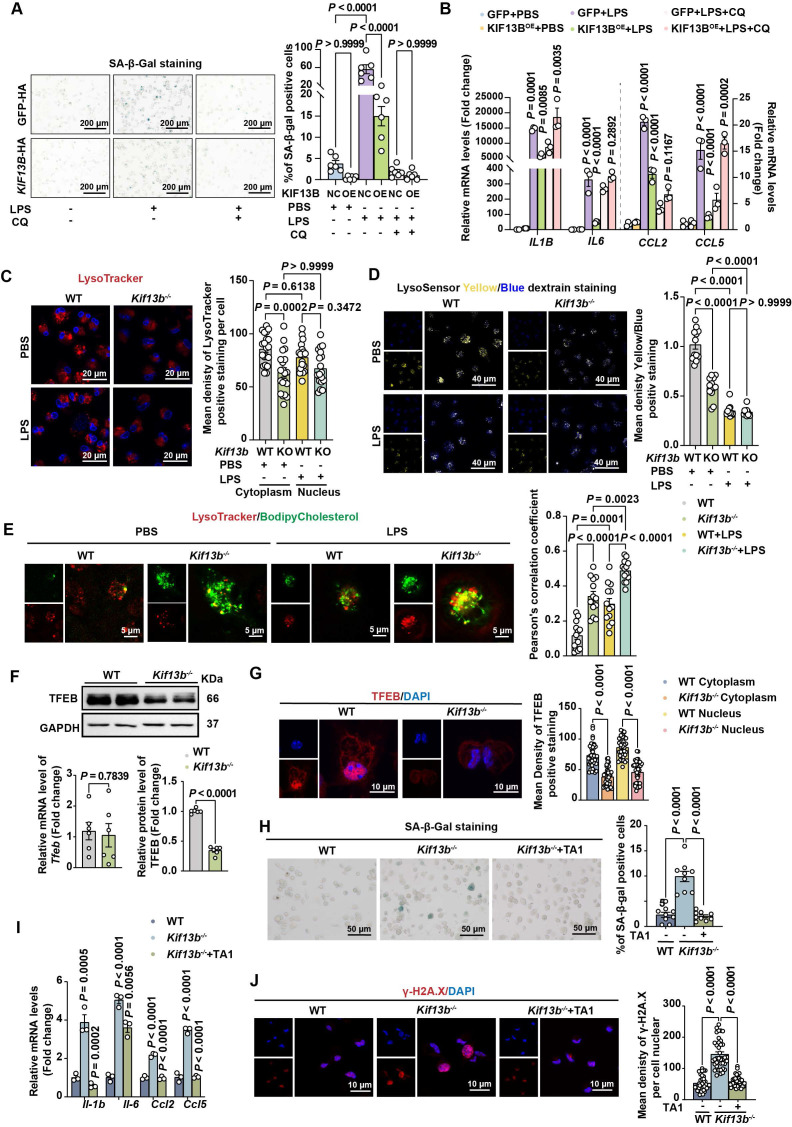
** KIF13B maintains lysosomal function to inhibit macrophage senescence *in vitro*. (A-B)** WT mouse BMDMs were infected with LV-GFP-HA (negative control, NC) or LV-* KIF13B* -HA overexpressing* KIF13B* (overexpression, OE), followed by treatment with PBS or 1 μg/ml LPS for 24 h ± chloroquine (CQ) pretreatment. **(A)** SA-β-gal staining (representative images, n = 6/group). **(B)** qPCR analysis of SASP-related genes (n= 3 /group).** (C-E)** Primary BMDMs from 8-week-old male WT and *Kif13b^-/-^* mice stimulated with PBS or 10 ng/ml LPS (n = 3 independent experiments/group)**. (C)** Lysotracker (red) and DAPI (blue) staining (images and lysosomal density quantification). **(D)** Lysosensor staining (images and Yellow/Blue ratio quantification for pH measurement). **(E)** Bodipy-cholesterol uptake (images and Pearson's coefficient for lysosome-cholesterol colocalization). **(F)** qPCR and Western blot analysis of TFEB mRNA and protein expression levels in primary mouse BMDMs from WT and *Kif13b^-/-^* mice (n = 6/group), respectively. **(G)** Immunofluorescence of TFEB (red) with DAPI (images and quantification of cytoplasmic/nuclear density, n=6 experiments). **(H-J)** Primary mouse BMDMs were extracted from 8-week-old female WT and *Kif13b^-/-^* mice and then TFEB activator 1 (TA1) was used to treat *Kif13b^-/-^* macrophages.** (H)** SA-β-gal staining (n= 9 /group).** (I)** SASP gene expression (n= 3 /group). **(J)** γ-H2A.X staining (images and nuclear density quantification, n = 6 experiments). Data were presented as mean ± SEM. Data **A-E** and **G** were analyzed by two-way ANOVA. Data **F** were analyzed by unpaired Student's t-test. Data **H-J** were analyzed by one-way ANOVA with Bonferroni post-hoc tests.

Since KIF13B executed beneficial functions in cellular senescence and inflammation, to determine whether *KIF13B* overexpression could counteract senescence and inflammation, we constructed a lentiviral vector overexpressing *Kif13b* (LV-* KIF13B*-HA) to infect WT mouse BMDMs. Overexpression of *KIF13B* significantly reduced the level of senescence and decreased the mRNA expression of pro-inflammatory genes in macrophages, exclusively in LPS-induced pro-inflammatory macrophages (Figure 6A-B).

Emerging evidence implicates that lysosomal dysfunction in cellular senescence 28. Our transcriptomic analysis revealed that genes associated with lysosomal pathway were highly enriched and their expression was upregulated upon *Kif13b* deletion (Figure S4A). To test whether KIF13B's beneficial effects depended on lysosomal activity, we employed the lysosomal inhibitor chloroquine. In WT mouse BMDMs pretreated with chloroquine, overexpression of *KIF13B* failed to suppress macrophage senescence and inflammation (Figure 6A-B). Moreover, Chloroquine-treated WT BMDMs showed no additional senescence exacerbation upon *Kif13b* deletion (Figure S4B), indicating that lysosomal function is a key mediator required for the regulatory role of KIF13B.

Next, to define the role of KIF13B in lysosomal regulation, we analyzed lysosomal integrity and activity in BMDMs. Lysotracker staining revealed a significant reduction in lysosome number in *Kif13b*-deficient cells compared to WT controls, whereas LPS stimulation failed to worsen this deficit in *Kif13b*-deficient cells (Figure 6C), suggesting that lysosomal dysfunction is a major consequence of *Kif13b* deficiency. In addition, we assessed lysosomal activity by monitoring lysosomal pH using Lysosensor and found that lysosomal activity was markedly decreased in *Kif13b*-deficient macrophages compared to controls treated with PBS (Figure 6D). However, LPS stimulation abolished this difference, likely due to lysosomal stress overriding KIF13B-dependent regulation (Figure 6D). Additionally, *Kif13b* deficiency diminished the mRNA abundance of vacuolar *ATPases* (V-ATPases), an essential regulator responsible for lysosomal proton transport (Figure S4C). Collectively, these findings demonstrate that knocking out *Kif13b* reduces the number of macrophage lysosomes and increases lysosomal pH, thus disrupting proper lysosomal functions.

Based on the evidence demonstrating a link between lysosomal cholesterol accumulation and SASP phenotype 29, we determined whether KIF13B regulated lysosomal cholesterol homeostasis. BODIPY-cholesterol labeling in BMDMs revealed that more lysosomal cholesterol content was detected in *Kif13b*-deficient cells under the PBS treatment condition, and *Kif13b* deletion further exacerbated lysosomal cholesterol accumulation in LPS-treated macrophages (Figure 6E).

TFEB is implicated in aging and age-related diseases 30. We investigated whether TFEB mediated KIF13B's effects on lysosomal homeostasis. Interestingly, *Tfeb* mRNA expression remained unchanged in both WT and *Kif13b^-/-^* BMDMs (Figure 6F). However, using confocal technique and Western blot we found that TFEB protein levels, particularly in the nucleus, were dramatically reduced in *Kif13b*-deficient macrophages (Figure 6F-G), indicating impaired nuclear translocation. Moreover, we observed that TA1, a TFEB agonist, significantly reversed SASP phenotype in *Kif13b*-deficient macrophages, with fewer SA-β-gal-positive cells and less DNA damage, nearly close to WT control cells (Figure 6H-J). These data demonstrated TFEB as a critical downstream effector of KIF13B, mediating its anti-senescent and anti-inflammatory roles in macrophages.

### KIF13B stabilizes TFEB via USP9X-mediated deubiquitination

**Figure 7:**
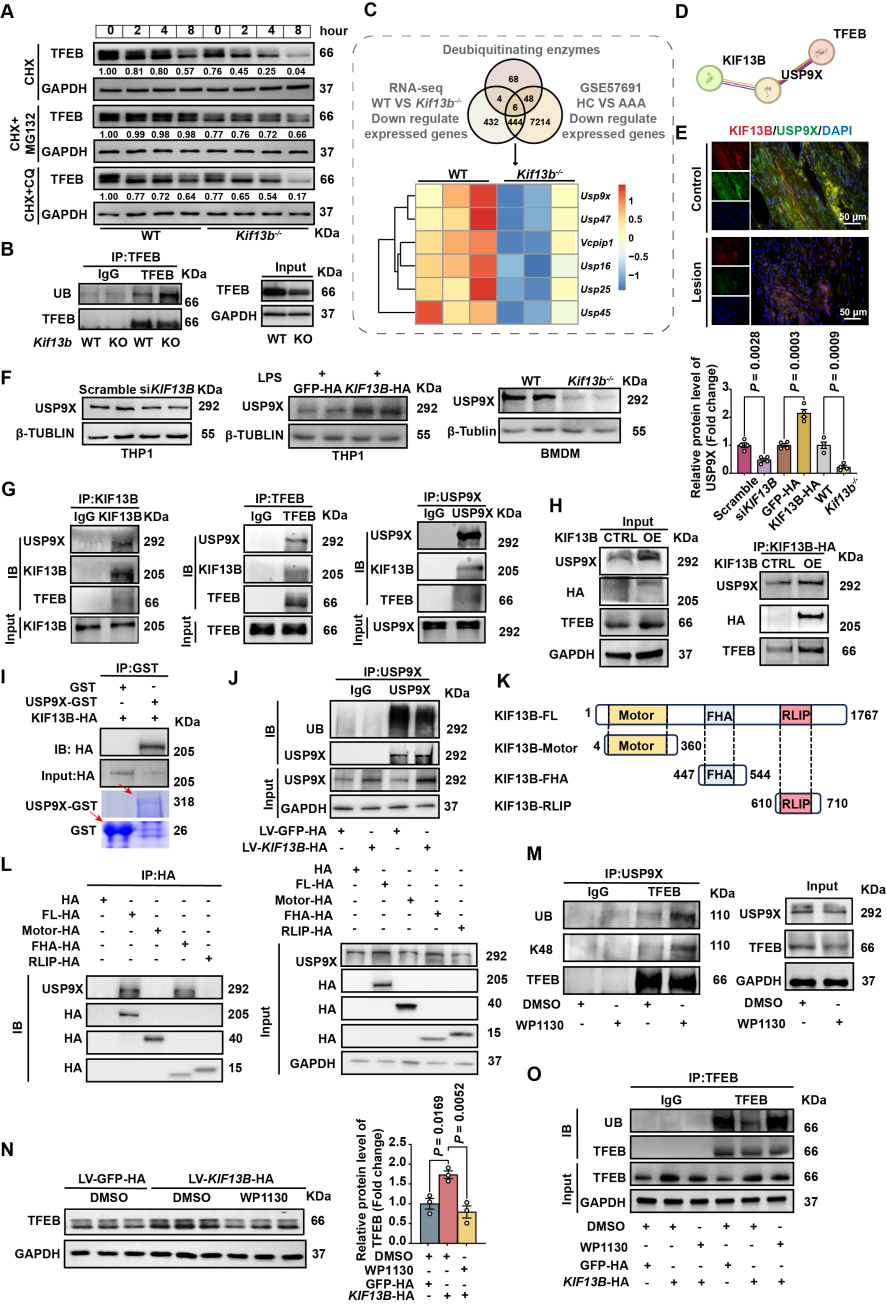
** KIF13B stabilizes TFEB via USP9X-mediated deubiquitination. (A)** TFEB protein stability was assessed by western blot in macrophages treated with PBS, 10 µM MG132 (proteasome inhibitor), or 10 µM chloroquine (lysosomal inhibitor) in the presence of 50 µg/ml cycloheximide (CHX) at indicated time points. **(B)** Ubiquitination status of TFEB was evaluated by co-immunoprecipitation in primary BMDMs (n = 3/group). **(C)** Integrated transcriptomic analysis identified six significantly downregulated deubiquitinating enzymes in both* Kif13b^-/-^* BMDMs and human AAA tissues (heatmap presentation). **(D)** The HDOCK database suggests that USP9X can bind to KIF13B. **(E)** Immunofluorescence imaging demonstrated co-localization of USP9X (green) and KIF13B (red) in human AAA lesions versus adjacent healthy tissue (n = 3/group). **(F)** USP9X expression was quantified by western blot in THP1-derived macrophages following *KIF13B* knockdown or overexpression, primary WT versus *Kif13b^-/-^* BMDMs (n = 4/group in each condition). **(G-I)** Protein interactions were confirmed through endogenous **(G)**, semi-exogenous** (H)** co-IP assays in primary BMDMs and *in vitro* GST pull-down assays using recombinant proteins. (**J**) Ubiquitination status of USP9X was evaluated by co-immunoprecipitation in primary BMDMs (n = 3/group). **(K)** Schematic of human full-length KIF13B and its three functional domains (Motor, FHA, RLIP). **(L)** Protein interactions between USP9X and KIF13B or its different functional domains were confirmed through semi-exogenous co-IP assays in THP1-derived macrophages **(M)** TFEB ubiquitination was measured following treatment with 5 µM WP1130 (deubiquitinase inhibitor). **(N-O)** TFEB protein levels**(N)** and ubiquitination status **(O)** were analyzed in *KIF13B* -overexpressing BMDMs with DMSO or WP1130 treatment (n = 3/group). Data represent mean ± SEM. **F** was analyzed by unpaired Student's t-test. **N** was analyzed by one-way ANOVA with Bonferroni correction.

As KIF13B localizes to the cytoplasm and does not directly regulate *Tfeb* transcription, we hypothesized it might modulate TFEB at the post-translational level. siRNA-mediated *Kif13b* knockdown in THP1-derived macrophages accelerated TFEB degradation under cycloheximide (CHX)-mediated translational blockade. By treating cells with the proteasome inhibitor MG132 or the lysosomal inhibitor chloroquine, respectively, we found that TFEB was mainly degraded through the proteasomal ubiquitination pathway (Figure 7A). Additionally, co-immunoprecipitation (co-IP) confirmed enhanced TFEB ubiquitination in *Kif13b*^-/-^ macrophages (Figure 7B).

Next, to identify the potential ubiquitination enzymes responsible for TFEB degradation regulated by KIF13B, we integrated transcriptomic data primary mouse BMDMs and human AAA tissues, revealing six downregulated deubiquitinating enzymes (DUBs), including *Usp9x, Usp45, Vcpip1, Usp47, Usp16* and *Usp25.* Among these, USP9X was the only candidate predicted by the HDOCK database to functionally interact with KIF13B with a high confidence score of 0.9186 (Figure 7C-D). Our immunofluorescence results showed a co-localization of KIF13B and USP9X in healthy aortic walls, which was abolished in AAA lesions (Figure 7E). Furthermore, Western blot data showed that siRNA-mediated *Kif13b* knockdown in THP1-derived macrophages reduced USP9X protein levels (Figure 7F). Conversely, LV-mediated overexpression of *KIF13B* in THP1-derived macrophages led to an increase in USP9X, which were consistently observed in BMDMs isolated from *Kif13b^-/-^* mice (Figure 7F). Importantly, co-IP revealed physical interaction between KIF13B, TFEB and USP9X (Figure 7G-H). Moreover, KIF13B was further validated to directly interact with USP9X through *in vitro* binding assays, demonstrating the effect on USP9X protein stability by suppressing ubiquitination-dependent degradation (Figure 7I-J).

To clarify the structure-function relationship of KIF13B and USP9X stabilization, a domain-specific transfection strategy was employed in THP1-derived macrophages by overexpressing full-length KIF13B and its discrete functional domains. It is noteworthy that USP9X only bound to fu full-length KIF13B or its FHA domain, concomitant with enhanced USP9X protein levels (Figures 7I-J). Consistently, the FHA domain alone possessed the anti-senescence capacity of full-length KIF13B, significantly reducing LPS-induced senescence markers in macrophages (Figure S5A). To confirm that USP9X may act as a deubiquitinating enzyme for TFEB, we treated mouse BMDM with the inhibitor of USP9X, WP1130 31, which reduced USP9X and TFEB protein levels while increased K48-linked ubiquitination of TFEB (Figure 7M), consistent with proteasomal degradation. Additionally, the elevation of TFEB protein levels by *KIF13B* overexpression in BMDMs was abolished in the presence of WP1130 treatment, an effect that was mechanistically linked to altered ubiquitination dynamics (Figure 7N-O). Taken together, our data demonstrated that the FHA domain of KIF13B specifically mediates stabilization of USP9X protein, which subsequently deubiquitinates TFEB to suppress macrophage senescence and inflammatory responses.

### Reconstitution of macrophage KIF13B or senolytic therapy effectively attenuates AAA progression *in vivo*

**Figure 8:**
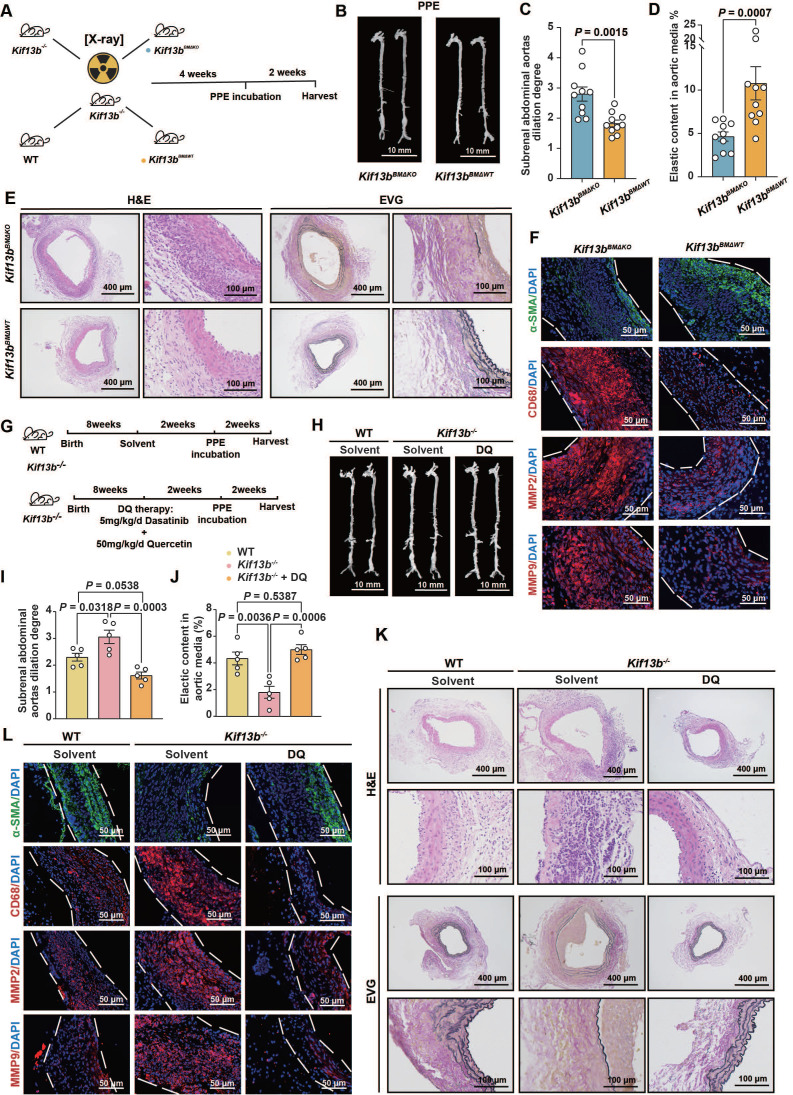
** Reconstitution of macrophage KIF13B or senolytic therapy effectively attenuates AAA progression *in vivo*. (A-F)** Eight-week-old male *Kif13b^-/-^* mice underwent bone marrow transplantation (BMT) from either *Kif13b^-/-^* or WT donors, followed by PPE-induced AAA modeling. **(A)** Study design (n = 10/group).** (B)** Representative images of the macroscopic features. **(C)** Quantitative analysis of the ratio of the maximal diameter of the AAA to the untreated normal aorta. **(D)** Residual elastic fiber area relative to vessel wall area. **(E)** Representative images of HE staining and EVG staining in each group. **(F)** Representative immunofluorescence staining images of α-SMA (green), CD68 (red), MMP2 (red) and MMP9 (red) expression levels in each group.** (G-L)** WT and *Kif13b^-/-^* mice received dasatinib (5mg/kg/d) + quercetin (50mg/kg/d) or vehicle for 2 weeks pre-PPE induction (n = 5/group). **(G)** Study design. **(H)** Representative images of the macroscopic features. **(I)** Quantitative analysis of the ratio of the maximal diameter of the AAA to the untreated normal aorta. **(J)** Residual elastic fiber area relative to vessel wall area. **(K)** Representative images of HE staining and EVG staining in each group. **(L)** Representative immunofluorescence staining images of α-SMA (green), CD68 (red), MMP2 (red) and MMP9 (red) expression levels in each group. Data were presented as mean ± SEM. Data **C, D** was analyzed by un-paired student t test. Data **I, J** was analyzed by one-way ANOVA with Bonferroni correction.

Based on the confirmed observations that *Kif13b* deficiency in macrophages triggered the exacerbation of AAA as described above, we sought to develop an intervention for mitigating the AAA process by reconstituting KIF13B in mouse macrophage. *Kif13b*^-/-^ mice underwent bone marrow transplantation (BMT) and then received bone marrows isolated from either WT or *Kif13b*-deficient mice (Figure 8A). The results showed that transplantation of WT bone marrows significantly attenuated the severity degree of PPE-induced AAA expansion and elastic fiber degradation compared to *Kif13b^-/-^* mice transplanted with* Kif13b^-/-^* bone marrows (Figure 8B-D). Consistently, the phenotypes of AAA with accelerated extracellular matrix degradation, loss of contractile smooth muscle cells, macrophage infiltration and MMP secretion in PPE-infused* Kif13b^-/-^* mice were largely reversed by transplanting WT bone marrows (Figure 8E-F). Furthermore, to clarify the impact of senescence on AAA development in the context of *Kif13b* deficiency, we performed senolytic therapy on *Kif13b^-/-^* mice. In agreement with the senolytic results *in vitro*, oral administration of dasatinib and quercetin to *Kif13b^-/-^* mice significantly reversed AAA severity when compared to solvent-treated *Kif13b^-/-^* mice (Figure 8G-L), further suggesting that restoring myeloid KIF13B or eliminating senescent cells mitigates AAA progression caused by *Kif13b* deficiency, positioning KIF13B as a key protector against macrophage senescence-driven vascular pathology.

## Discussion

AAA is a life-threatening disease with high morbidity and mortality worldwide; however, the therapeutic treatment is still lacking to date because the pathogenesis underlying this devastating disorder is complicated and remains elusive. In the present study, our results have elucidated pathogenic mechanisms linking the sustained senescence and proinflammation of macrophages to AAA formation. Meanwhile we also identified KIF13B as a key molecule responsible for maintaining proper lysosomal function to restrict senescence and inflammatory response in macrophages. First, our investigation revealed a significant reduction in KIF13B expression levels within aortic aneurysm lesions from both human patients and multiple mouse AAA models. Global inactivation of *Kif13b* or macrophage specific deletion of *Kif13b* exacerbated the development of aortic aneurysm in PPE or ANG II-infused mouse models. Furthermore, mechanistic studies revealed that depleting *Kif13b* elicited accelerated senescence of macrophages accompanied with a phenotypic switch of M2 to M1 due to abnormal cholesterol accumulation in lysosomes and impaired TFEB-dependent lysosomal biogenesis. KIF13B mediated the stability of TFEB at the protein level by interacting with USP9X, a critical deubiquitinating enzyme (DUB), thereby enhancing TFEB deubiquitination and elevating its cellular levels. Supplementation of TFEB agonist also reversed the deleterious effects caused by *Kif13b* deficiency in cultured macrophages *in vitro*. Finally, macrophage-specific restoration of KIF13B through BMT or senolytic therapy exhibited a pronounced protective effect on AAA progression in *Kif13b^-/-^* mice. These findings demonstrate that macrophage KIF13B acts as a protective factor of AAA pathogenesis.

KIF13B, the largest member of kinesin-3 family, possesses multiple functions, including cellular signaling transduction, receptor trafficking and cell division 15,32-34, and its deficits are associated with neurodegeneration, developmental defect and cancer 18,35,36. Recently, our work discovered that hepatic KIF13B expression levels were negatively correlated with MAFLD progress and KIF13B orchestrated mitochondrial function and de novo lipogenesis (DNL) to maintain liver energy homeostasis by activating AMPK pathway, suggesting that KIF13B plays a critical role in hepatic lipid metabolism and the related metabolic disease 21. To our knowledge, MAFLD has been reported to contribute to CVD through the mechanisms related to metabolic dysregulation, chronic inflammation, and oxidative stress 22. Recent evidence from a UK-based prospective cohort study further supports a positive association between MAFLD and AAA 23. Interestingly, while whole body *Kif13b* deficiency resulted in aggravated AAA expansion in both PPE and ANG II-infused mouse models, the latter caused more severity of AAA development and higher death rate than the former. This difference is likely due to the additional induction of hyperlipidemia in ANG II-infused mice through AAV8-PSCK9^D337Y^ delivery and Western diet feeding. Consistently, two independent studies led by Roychowdhury and Lu have reported that PSCK9 and triglyceride rich lipoproteins could be potential therapeutic targets for the treatment of AAA 5,9, further supporting the concept that hyperlipidemia is a risk factor of AAA pathogenesis.

It is noteworthy that multiple cell types contribute to the pathogenesis of AAA, yet how *Kif13b* deficiency exacerbated AAA expansion remained unknown in the whole-body knockout mice. To address this, using single-cell RNA sequencing technology and bioinformatic analyses, we identified that macrophages were enriched and accumulated in the aortic lesions in *Kif13b^-/-^* mice, in which more M1-like cells marked with *CCR2, CD80*, and *CD86* and less M2-like cells marked with *CD206* and *TREM2* were observed. These findings are consistent with the previous observations 37, suggesting that macrophages migrate into the injured sites and then contribute to the inflammatory response. Importantly, although VSMC phenotypic switching is a well-established characteristic of AAA, we unexpectedly found that VSMC-specific *Kif13b* depletion did not influence AAA formation. In contrast, macrophage-specific *Kif13b* knockout mice exhibited increased susceptibility to PPE-induced AAA. These results indicate that macrophages rather than VSMCs serve as the primary cellular drivers of AAA pathogenesis in the context of *Kif13b* deficiency. Therefore, loss of *Kif13b* in macrophages was sufficient to promote AAA development independently of plasma lipid level alterations.

Inflammation of the vascular wall has long been recognized as a key trigger for AAA 38. Chronic inflammation, one of the twelve hallmarks of aging, is inextricably linked to the aging process 26,39. Senescent cells that possess highly metabolic activities can secrete pro-inflammatory factors, termed SASP, to promote chronic inflammation, which in turn drives healthy cells into the senescence process 40. Over the past decade, vascular cell senescence has been progressively linked to AAA pathogenesis, and significant progress has been made particularly in the field of the therapeutic targeting of smooth muscle cell senescence for the treatment of AAA disease 41-43. However, the role of macrophage senescence in AAA pathogenesis remains poorly understood, despite linking immune dysfunction, which contributes to age-related and autoimmune diseases, to the aberrant accumulation of senescence-like macrophages 44. Remarkably, our *in vitro* data demonstrated that *Kif13b*-deficient macrophages spontaneously developed senescent characteristics, with elevated SA-β-gal activity and activated SASP, even without proinflammatory intervention such as LPS stimulation. These cells also exhibited enhanced DNA damage responses and upregulated cyclin-dependent kinase inhibitors (CDKIs), resulting in G1 phase cell cycle arrest. Senescent macrophages lacking *Kif13b* expression migrated to and accumulated in the vascular wall, impairing vascular homeostasis through enhanced inflammatory responses, then leading to accelerated MMP-mediated extracellular matrix degradation and ultimately promoted AAA progression.

Importantly, accumulating evidence suggests that the integrity and function of lysosomes significantly decline in various diseases and during aging 45-47. Ruckenstuhl et al. have positioned the lysosome as a central cellular hub for the control of aging 28. A recent study has also confirmed that abnormal cholesterol accumulation in the lysosome is a hallmark of lysosomal dysfunction linked to aging-related inflammation 29. Consistently, our results also showed that *Kif13b*-deficient BMDMs exhibited a reduced number of lysosomes, abnormally elevated lysosomal pH reflecting impaired enzymatic activity, and substantial cholesterol accumulation within lysosomes. Of note, it has been documented that when lysosomes are damaged, TFEB, a key transcription factor required for lysosomal biogenesis, undergoes nuclear translocation to activate the transcription of lysosome-associated genes 48. Dysregulated TFEB has been detected in patients with aneurysms, which is highly associated with smooth muscle cell apoptosis and macrophage inflammation, but the underlying molecular mechanisms are still missing 49,50. In the present study, TFEB was identified as a central downstream target of KIF13B in macrophages to mediate the regulation of macrophage senescence by KIF13B. Interestingly, rather than being able to promote nuclear translocation of TFEB, KIF13B also upregulated the protein level of TFEB by inhibiting its degradation via the proteasomal ubiquitination pathway. Through a comprehensive intersection analysis of transcriptomics data and GEO database combined with predicted functional interaction by STRING software, we identified USP9X, one of the deubiquitinating enzyme family members as a crucial modulator involved in KIF13B-mediated TFEB regulation. Interestingly, several studies have reported that USP9X mainly controls cell adhesion and polarity, which has been implicated in neurodegenerative disease and tumor 51,52. However, recent investigation led by Wang and colleagues discovered that USP9X executed protective function in the development of atherosclerosis 53. Similarly, our results also revealed a significant reduction in USP9X expression levels in the aortic lesions of AAA patients. We identified TFEB as a downstream target of USP9X in macrophages, wherein KIF13B suppresses TFEB ubiquitination and attenuates senescence through interacting with USP9X. Furthermore, BMT with WT bone marrows to* Kif13b^-/-^* mice or administration of senolytic therapy could largely reverse AAA progress *in vivo*, demonstrating that targeting macrophage KIF13B represents a novel therapeutic strategy for AAA. Given the intricate regulatory network governing the dynamic balance between ubiquitination and deubiquitination, our findings reported in the present study, while identifies that the FHA domain of KIF13B binds to USP9X and then maintains its protein stability, thereby enhancing USP9X protein expression, cannot fully elucidate the precise regulatory mechanism underlying this stabilization at the current stage and future investigations are warranted to solve this issue.

In summary, our current study provides the first evidence that KIF13B exerts a protective effect against AAA pathogenesis. We elucidated a novel molecular mechanism wherein KIF13B stabilizes TFEB through promoting USP9X-mediated deubiquitination to maintain lysosome homeostasis, thus mitigates macrophage senescence and inflammation. These findings not only identified an inhibitory role of macrophage-derived KIF13B in AAA progress, but also highlighted the potentially therapeutic strategies for the treatment of AAA disease.

## Supplementary Material

Supplementary materials and methods, figures and tables.
