# Supplementary material for: Macrophage-derived KIF13B interacts with USP9X to attenuate abdominal aortic aneurysm development by potentiating TFEB stability
Source: Theranostics. 2025 Aug 16;15(17):9114–30. doi: 10.7150/thno.118958 (PMC12439347; doi:10.7150/thno.118958)
Supplement: Supplementary file 1 — Supplementary materials and methods, figures and tables. [file thnov15p9114s1.pdf]

# Supplemental Material

## Supplementary Methods

### Abdominal Aortic Aneurysm (AAA) Animal Models

*Kif13b*$^{-/-}$ mice, wild-type (WT) control mice, conditional knockout (myeloid-specific, *Lyz2Kif13b*$^{f/f}$ and smooth muscle cell-specific, *Sm22Kif13b*$^{f/f}$) mice and *Kif13b*$^{f/f}$ littermates were used in the study. All animals were housed at 24 ± 2°C and 40 ± 5% humidity with a 12 h light/dark cycle, and were allowed to drink and eat ad libitum without specific instructions. To establish a porcine pancreatic elastase (PPE, HY-P2974, MCE, America) induced AAA mouse model, 8-10-week old male mice were selected and under general anaesthesia, the infrarenal abdominal aortic segments were isolated and wrapped in gauze pre-impregnated with 10 mg/ml PPE for 40 min. After removal of the gauze, the abdominal cavity was washed with saline before suturing. Fourteen days after surgery, the mice were anaesthetized and the aorta was harvested. The diameter of the infrarenal AAA and the diameter of the untreated normal vessel were measured using Image J, and the ratio of the two was used as the multiplicity of AAA expansion.

To establish a PCSK9/angiotensin II (ANG II)-induced AAA model, 14 *Kif13b*$^{-/-}$ mice and 17 WT mice were used. Adeno-associated virus 8 (AAV8) expressing PCSK9D377Y was injected intraperitoneally into 10- to 12-week-old mice at a dose of 2E11 genome copies per mouse, followed by feeding a Western diet (WD, 22.6% protein, 20.1% fat, 45.4% carbohydrate, 1.25% cholesterol, 40% fat for energy; D12108C,Research Diets, America) to induce hyperlipidemia. Three weeks after AAV injection, mice were anaesthetized and Ang II (1,000 ng/kg/min; HY-13948, MCE, America) or saline (0.9% NaCl) was infused subcutaneously in the dorsal neck using a micro-osmotic pump ( 2004, Alzet). After 28 days, the mice were anaesthetized and the aortas were harvested. Necropsy was performed on all mice before the study endpoint, and the criterion for aneurysm rupture was the presence of a thrombus outlet outside the expanded aortic wall epithelium. Maximum aortic diameters for each segment were measured separately using Image J, including the aortic root, ascending aorta, descending aorta and abdominal aorta. Aortic dissection was excluded from the analysis of aortic diameter but included in the analysis of aneurysm incidence.

We performed bone marrow transplantation to study the therapeutic effect of KIF13B in macrophage on AAA: The mice were lethally irradiated (8 Gy with X-ray), then injected IV (tail vein) with 1E7 bone marrow cells from donor mice for bone marrow transplantation as previously described. Briefly, one day before and 2 days after the transplantation, mice were fasted and fed with DMEM culture medium intragastrically.

During the recovery phase of 4 weeks, mice were fed with water containing Penicillin and Streptomycin.

*Kif13b*$^{-/-}$ mice and WT mice were used as bone marrow donors to reconstitute lethally irradiated *Kif13b*$^{-/-}$ mice.

Dasatinib and quercetin treatment in mice also used WT mice, *Kif13b*$^{-/-}$ male mice, which were first gavaged with dasatinib (5mg/kg/d, HY-10181, MCE, America) and quercetin (50mg/kg/d, HY-18085, MCE, America) or solvent for 2 weeks, and then established PPE-induced AAA mouse model.

**Cell Culture**

Primary bone marrow-derived macrophages (BMDMs) were extracted from WT and *Kif13b*$^{-/-}$ mice. Cells were flushed from the tibia and femurs of 6- to 8-week-old WT or *Kif13b*$^{-/-}$ mice, and then were cultured in DMEM supplemented with 10% FBS (FS301-02, TransGen, China), and 1% penicillin-streptomycin and stimulated with 25 ng/mL mouse macrophage colony-stimulating factor 1 (M-CSF ; 315-02, Peprotech, America) for 6 days.

THP1 cells were a gift from Professor Yan Zhang at Peking University. THP1 cells were treated with 100ng/ml PMA (P6741, Solarbio, China) for 48h to induce the differentiation of Thp1 into macrophages, and then cultured in 1640 medium supplemented with 10% FBS, 100 units/mL penicillin and 100 μg/mL streptomycin. All cells were maintained in a humidified 5% CO2 incubator at 37°C.

LPS was used to induce transdifferentiation of macrophages into pro-inflammatory macrophages. In primary BMDMs from WT and *Kif13b*$^{-/-}$ mice, we used 10 ng/ml LPS (L2630, Sigma, Germany). In cells infected with LV-GFP-HA and LV-*KIF13B*-HA, we used 1 μg/ml LPS (L2630, Sigma, Germany). 1μM TFEB activator 1 was used to stimulate macrophages for 12 h to promote TFEB nuclear translocation.

To investigate the stability of TFEB in primary mouse BMDMs, we used the protein synthesis inhibitor cycloheximide (CHX) (50 μg/ml; HY-12320, MCE, America), the proteasomal degradation inhibitor MG132 (10 μM; HY-13259, MCE, America), the lysosomal degradation inhibitor CQ (10 μM; HY-17589A, MCE, America) to treat macrophages.

To investigate the role of USP9X in the regulation of TFEB ubiquitination, we treated macrophages with the USP9X inhibitor WP1130 (5 μM; HY-13264 , MCE, USA).

**Analysis of Blood Pressure and Plasma Lipid Levels**

Mouse blood pressure was measured using a non-invasive tail collar system (CODA®; Kent Scientific, America). Plasma triglyceride and total cholesterol levels

were determined using kits purchased from BIOSINO BIO-TECHNOLOGY & SCIENCE INC (Beijing, China).

**Preparation and Application of Lentivirus**

Lentiviral vectors (15 mg), along with pMDLg/pRRE, RSV/Rev, and VSV-G (5 mg each), were co-transfected into 293T cells using the calcium phosphate ($Ca_3(PO_4)_2$) method in 10-cm dishes. The cells were incubated for 12-16 h, after which the medium was replaced. Two days post-transfection, the viral supernatant was collected and filtered through 0.45 μm filters (SLHP033RB, Millipore, America). The filtered supernatant was concentrated to 1e8 TU through ultracentrifugation, to be used for in vitro experiments. Lentiviruses were either used immediately or stored at -80°C for future use.

**RNA Interference**

The si*KIF13B* and scramble sequences were synthesized by Sangon Biotech (Shanghai, China). The specific siRNA sequences are listed in the table S3. THP1-derived macrophages with a density of 3E6 cells per well were passaged in a 6-well plate with Opti-MEM medium and transfected with siRNA using lipofectamine RNAiMAX (13778150, Invitrogen, American) as described by the manufacturer. Cells were harvested 72 h after transfection to analyze knockdown efficiency.

**Plasmid Transfection**

HA-tagged expression constructs including full-length KIF13B plasmid (FL-HA, amino acids 1-1767), KIF13B motor domain plasmid (Motor-HA, 4-360), KIF13B FHA domain plasmid (FHA-HA, 447-544), and KIF13B RLIP domain plasmid (RLIP-HA, 610-710) were synthesized by Sangon Biotech (Shanghai, China). Transfection was performed using Lipofectamine 3000 (L3000015, Invitrogen, American) according to the manufacturer's protocol in THP1-derived macrophages. Cells were harvested 72 h after transfection for subsequent analyses.

**Protein Purification and GST Pull-down Assay**

HA-fusion proteins were obtained from HEK293A cells overexpressing KIF13B-HA, incubated with Anti-HA Affinity Gel (Beyotime, P2287) for 4-6 h, and eluted using HA Peptide (Beyotime, P9808). USP9S-GST plasmid was obtained from MiaoLingPlasmid, China. GST-fusion proteins purified from *E. coli* BL21 were immobilized on Glutathione-Sepharose 4B beads (GE Healthcare; 50 μL slurry) for 2-4 h. Purified HA-fusion proteins were then mixed with GST-fusion protein-bound beads

and incubated overnight at 4°C on an end-over-end rotator. After three washes with ice-cold RIPA buffer, bound complexes were eluted in SDS-PAGE loading buffer by boiling (95°C, 10 min) and analyzed via Western blot with target-specific antibodies.

**Immunofluorescent Staining**

Paraffin-embedded sections (5 μm thickness) were deparaffinized and rehydrated, followed by antigen retrieval in EDTA retrieval solution, and then embedded in PBS containing 10% donkey serum and incubated with anti-KIF13B (SAB2101257, Sigma, Germany), anti-α-smooth muscle actin (α-SMA) (BM0002, Boster, China), anti-α-SMA (PA5-18929, Invitrogen, America), anti-Cluster of Differentiation 68 (CD68) (BA3638, Boster, China), anti- CD68 (ab955, ABCAM, UK), anti-matrix metalloproteinase 2 (MMP2) (BS-0412R, Bioss, China), anti-matrix metalloproteinase 9 (MMP9) (BS-0397R, Bioss, China), anti-phospho-histone H2A.X (S139) (γ-H2A.X) (P16104, Abmart, China), anti-ubiquitin-specific peptidase 9 X-linked (USP9X) (55054-1-AP, Proteintech, America) primary antibodies (1:200) were incubated overnight at 4°C. Normal isotype IgG (1:200, 3900; Cell Signaling Technology, America) (1:200, 5415; Cell Signaling Technology, America) was used as a negative control. After a rinse with PBS for 3 times, Alexa Fluor 555 or 647-conjugated secondary antibodies (1:200, ab150062, Abcam, Britain) (1:200, ab150107, Abcam, Britain) were incubated for 1 h at 37°C in the dark. Sections were made using an anti-fade mounting medium containing DAPI (ZLI9557, ZSGB-BIO, China) to stain cell nuclei.

Paste cell crawls were fixed with 4% paraformaldehyde for 10 min and then permeabilized with 0.2% (v/v) Triton X-100 in PBS for 10 min. After sealing in PBS containing 10% donkey serum, followed by an incubation with anti-KIF13B (SAB2101257, Sigma, Germany), anti-phospho-histone H2A.X (S139) (γ-H2A.X) (P16104, Abmart, China), anti-TFEB (13372-1-AP. Proteintech, America), anti-USP9X (sc365353, Santacruz, America) primary antibodies (1:200) overnight at 4°C, respectively. Normal isotype IgG (1:200, 3900; Cell Signaling Technology, America; 5415; Cell Signaling Technology, America) was used as a negative control. After a rinse with PBS for 3 times, Alexa Fluor 555 or 647-conjugated secondary antibodies (1:200, ab150062, Abcam, Britain; ab150107, Abcam, Britain) were incubated in the dark at 37°C for 1 h. DAPI (C1005, Beyotime, China) was used to visualize cell nuclei.

Super-resolution fluorescence imaging was performed by an integrated fluorescence microscope BZ-X810 (KEYENCE, America) or a confocal microscope FV3000 (Olympus, Japan). Immunofluorescence staining was quantified using Image J.

**Histological Analysis**

Aortic tissues were harvested, perfused with PBS, fixed in 4% paraformaldehyde, dehydrated in 20% sucrose solution, and embedded in paraffin for serial sectioning. The prepared paraffin sections (5 μm thickness) were stained with HE (hematoxylin, HHS128-4L, Sigma, Germany; eosin, HT110232-1L, Sigma, Germany) to visualize the general morphology, and elastin van Gieson (EVG) (BA4083A, Baso, China) staining to assess the degree of elastin degradation. The SA-β-Gal staining kit (C0602, Beyotime, China) was used to detect the level of senescence in AAA tissues. The degree of elastin degradation was quantified by counting the number of elastin fiber breaks per vessel or by counting the proportion of residual elastin fibers to the area of the middle layer of the vessel wall using Image-Pro Plus 6.0. Bright-field imaging was performed by an integrated fluorescence microscope BZ-X810 (KEYENCE, America).

**RNA Isolation and Quantitative PCR (qPCR)**

Total RNA was extracted from tissues or cells using Trizol reagent (ET111-01-V2, TransGen, China), and then 5 μg of total RNA was used for cDNA synthesis using the kit (AT321-01, TransGen, China. qPCR was performed using Top Green qPCR SuperMix (AQ132-24, TransGen, China) and analyzed on a QuantStudio 3 instrument (Thermo Fisher Scientific). Glyceraldehyde-3-phosphate dehydrogenase (*GAPDH*) was used as a housekeeping gene. Primer information is provided in Table S4-S5.

**Protein Extraction and Western Blot**

Proteins were extracted from abdominal aorta and cells using pre-cooled RIPA buffer (DE301-1, Di-Ning, China) containing protease inhibitor (04693116001, Roche, Switzerland) and phosphatase inhibitor (4906837001, Roche, Switzerland). The protein concentration in the lysates was quantified using the BCA Protein Assay Kit (23225, Thermo Fisher Scientific, America). The lysates were mixed with supersampling buffer (P0015L, Beyotime, China) and denatured at 95°C for 10 min, followed by SDS-PAGE and nitrocellulose membrane transfer. The membranes were immersed in 5% BSA in TBST buffer (25 mM Tris, 137 mM NaCl, 2.7 mM KCl, 0. 075% Tween-20) for 1 h at room temperature and then incubated with anti-KIF13B (SAB2101257, Sigma, Germany), anti-P21 (p38936, Abmart, China), anti-P16$^{INK4a}$ (p42771, Abmart, China), anti-phospho-histone H2A.X (S139) (γ-H2A.X) (P16104, Abmart, China), anti-TFEB (13372-1-AP. Proteintech, America), anti-USP9X (55054-1-AP, Proteintech, America), anti-UB ( 58395, Cell Signaling Technology, America), anti-HA (66006-2-Ig, Proteintech, America), anti-GAPDH (60004-1-Ig, Proteintech, America), anti-β-Actin (T40104 Abmart, China), anti-β-Tublin (M30109, Abmart, China)

primary antibodies were incubated overnight at 4°C. The membrane was then washed three times with TBST and incubated in TBST configured with horseradish peroxidase-conjugated secondary antibody (ZSGB-BIO, China) containing 5% BSA at room temperature for 1 h. The target protein bands were visualised with enhanced chemiluminescence solution (36208ES, Yeasen, China) using the iBright Imaging System (CL1500, Invitrogen, America).

**Assessment of Cell Apoptosis**

Terminal deoxynucleotidyl transferase dUTP incision end labelling (TUNEL) kit (11684817910, Roche, Germany) was used to assess the level of apoptosis, and the nuclei were labelled with DAPI (C1005, Beyotime, China), which were visualized using the confocal microscope FV3000 (Olympus, Japan).

For senescence-associated β-galactosidase (SA-β-Gal staining) activity, the SA-β-Gal staining kit (C0602, Beyotime, China) was used to detect the level of senescence of the cells. Bright-field imaging was performed by an integrated fluorescence microscope BZ-X810 (KEYENCE, America). Image J was used to quantify the number of SA-β-Gal positive cells as a percentage of all cells in each field.

**Assessment of Cell Reactive Oxygen Species (ROS)**

$5X10^5$ cells were plated in 24-well plates per well and the H2DCFDA probe (HY-D0940, MCE, America) was used to detect cellular ROS levels according to the manufacturer's instructions. Fluorescence images were captured using BZ-X810 (KEYENCE, America) and analyzed using Image J.

**Assessment of Cell Lysosome Number and Function**

The LysoTracker probe (C1046, Beyotime, China) was used to treat cells according to the manufacturer's instructions, and the mean fluorescence intensity was used to assess the number of lysosomes in the cells. Lysosensor Yellow/Blue dextran probe (40768ES50, Yeasen, China) was performed to measure lysosomal pH, and the ratio of the mean fluorescence intensity of yellow fluorescence and blue fluorescence was used to assess changes in lysosomal pH. The degree of cholesterol accumulation in lysosomes was analyzed using Pearson correlation coefficients for co-localization of Bodipy-cholesterol and LysoTracker in cells that were treated with a bodipy-labelled cholesterol and LysoTracker. All fluorescence images were captured with BZ-X810 (KEYENCE, America) and analyzed with Image J or Fiji.

**Coimmunoprecipitation (Co-IP) Assay**

Cells were lysed at 4℃with RIPA buffer containing protease inhibitors. The cell lysate supernatant was obtained by high-speed centrifugation, protein concentration was measured and an equal amount of lysate was used for immunoprecipitation. Cells were immunoprecipitated with anti-HA agarose gel beads (P2287, Beyotime, China) at 4℃overnight, or with anti-TFEB (13372-1-AP. Proteintech, America), or with anti-USP9X (55054-1-AP, Proteintech, America), anti-IgG (AC005, ABclonal, China) antibodies and protein A/G agarose beads (R8281, Solarbio, China) at 4℃ overnight. The precipitates were then washed three times with Washing RIPA buffer and the immunocomplexes were eluted with 2x SDS-PAGE protein loading buffer for 5 min at 95℃. The immunocomplexes were then separated by SDS-PAGE.

**RNA microarray data analysis**

The public microarray data were collected from GEO database (GSE57691) by GEOquery (http://seandavi.github.io/GEOquery/) R packages. We firstly performed PCA analysis to detect and remove anomalous samples to get accurate DEGs. The R package limma was used to normalize data and get DEGs between control and AAA groups [1]. Gene expression matrix was percentile shift normalized first and further scaled by limma.

**RNA-Seq data analysis**

The RNA-Seq data were firstly used for a quality check and filtered by trim_galore (https://github.com/FelixKrueger/TrimGalore) and then mapped to GRCm38 reference sequence by Hisat2. We then used HTSeq to calculate the expression matrix [2]. The R package DESeq2 was applied to generate differential expressed genes (DEGs) [3]. We used GSEA package to perform GSEA analysis based on the stat value in the DESeq2 results. The KOBAS online analysis website was used to perform pathway enrichment analysis in KEGG and GOBP database [4].

**Gene Correlation analysis**

Pearson correlation analysis was performed between *KIF13B* and other genes in gene sets obtained from KEGG database based on RNA microarray data after TMM normalization(n = 53). Genes with p value < 0.5 were considered to be significantly associated with *KIF13B* . For each gene set, genes were sorted by absolute Pearson correlation coefficient and we further selected top30 genes to display in the network diagram.

**Single cell RNA-Seq analysis**

The python package Scanpy was used to perform up-stream single cell RNA-Seq analysis [5]. After filtering cells and genes, we used scVI package to remove batch effect and perform data embedding, followed by a manual selection of cell type markers to annotate cell types [6]. The cell proportion analysis was conducted by pertpy (https://github.com/scverse/pertpy) package. The macrophages were isolated for further down-stream analysis. The sctour package was used to perform cell trajectory and pseudo-time analysis [7]. The GSVA and GSEA analysis were performed by GSEApy package [8]. GSVA score reflected the pathway activity of anti-inflammatory and pro-inflammatory gene sets in WT and KO group macrophages. GSVA score ranged from -1 to 1, GSVA score > 0 means pathway activity increased and the closer the absolute value is to 1, the higher the activity. By comparing the GSVA score between WT and KO, we can evaluate the components change of macrophages.

**RNA microarray data analysis**

The public microarray data were collected from GEO database (GSE57691) by GEOquery (http://seandavi.github.io/GEOquery/) R packages. We firstly performed PCA analysis to detect and remove anomalous samples to get accurate DEGs. The R package limma was used to normalize data and get DEGs between control and AAA groups [1].

**RNA-Seq data analysis**

The RNA-Seq data were firstly used for a quality check and filtered by trim_galore (https://github.com/FelixKrueger/TrimGalore) and then mapped to GRCm38 reference sequence by Hisat2. We then used HTSeq to calculate the expression matrix [2]. The R package DESeq2 was applied to generate differential expressed genes (DEGs) [3]. We used GSEA package to perform GSEA analysis based on the stat value in the DESeq2 results. The KOBAS online analysis website was used to perform pathway enrichment analysis in KEGG and GOBP database [4].

**Single cell RNA-Seq analysis**

The python package Scanpy was used to perform up-stream single cell RNA-Seq analysis [5]. After filtering cells and genes, we used scVI package to remove batch effect and perform data embedding, followed by a manual selection of cell type markers to annotate cell types [6]. The cell proportion analysis was conducted by pertpy (https://github.com/scverse/pertpy) package. The macrophages were isolated for further down-stream analysis. The sctour package was used to perform cell

trajectory and pseudo-time analysis [7]. The GSVA and GSEA analysis were performed by GSEApy package [8]. GSVA score reflected the pathway activity of anti-inflammatory and pro-inflammatory gene sets in WT and KO group macrophages. GSVA score ranged from -1 to 1, GSVA score > 0 means pathway activity increased and the closer the absolute value is to 1, the higher the activity. By comparing the GSVA score between WT and KO, we can evaluate the components change of macrophages.

## References

1. Ritchie ME, Phipson B, Wu D, Hu Y, Smyth GK. limma powers differential expression analyses for RNA-sequencing and microarray studies. Nucleic Acids Res. 2015; 43.

2. Anders S, Pyl PT, Huber W. HTSeq—a Python framework to work with high-throughput sequencing data. Bioinformatics. 2015; 31: 166–9.

3. Love MI, Huber W, Anders S. Moderated estimation of fold change and dispersion for RNA-seq data with DESeq2. Genome Biol. 2014; 15: 550.

4. Bu D, Luo H, Huo P, et al. KOBAS-i: intelligent prioritization and exploratory visualization of biological functions for gene enrichment analysis. Nucleic Acids Res. 2021; 49: W317–25.

5. Wolf FA, Angerer P, Theis FJ. SCANPY: large-scale single-cell gene expression data analysis. Genome Biol. 2018; 19: 15.

6. Lopez R, Regier J, Cole MB, Jordan MI, Yosef N. Deep generative modeling for single-cell transcriptomics. Nat Methods. 2018; 15: 1053–8.

7. Li Q. scTour: a deep learning architecture for robust inference and accurate prediction of cellular dynamics. Genome Biol. 2023; 24: 149.

8. Fang Z, Liu X, Peltz G. GSEApy: a comprehensive package for performing gene set enrichment analysis in Python. Lu Z, Ed. Bioinformatics. 2023; 39: btac757.

## Supplementary figures and figure legends

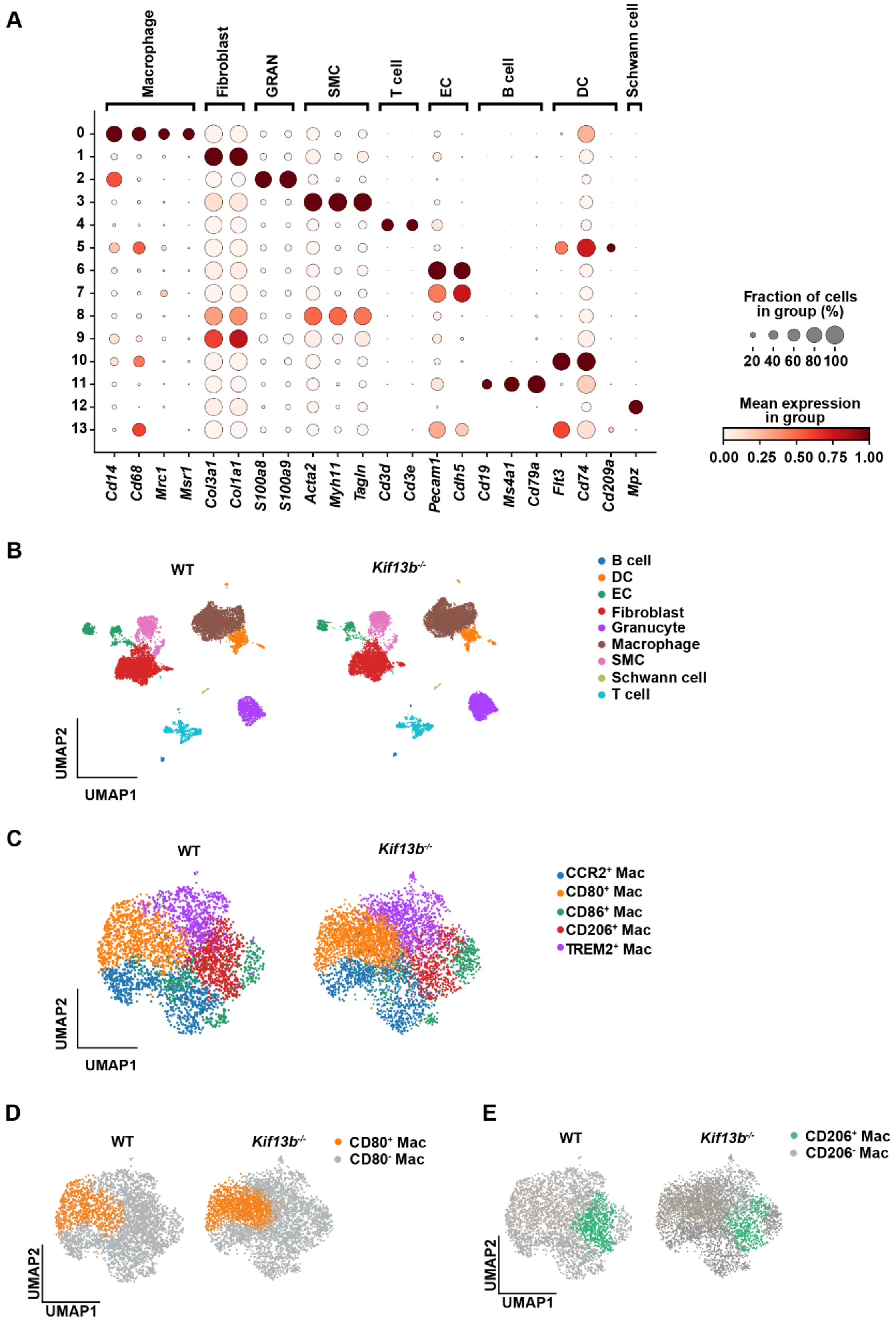


**Fig S1.** ***Kif13b*** **deletion elicits a phenotypic switch of M2 to M1 macrophages in AAA.**

**(A)** Mean expression and fraction of cell marker genes in all cell types. Expression level was scaled between each cluster. **(B)** UMAP plot depicting nine distinct cell clusters in AAA tissues from WT and *Kif13b*$^{-/-}$ mice samples separately, color-coded by cell type. **(C)** UMAP plot highlighting five macrophage subpopulations in AAA tissues from WT and *Kif13b*$^{-/-}$ mice samples separately, color-labeled for differentiation. **(D)** Distribution of CD80$^{+}$ macrophages (orange) and CD80$^{-}$ macrophages (grey) between the two groups. **(E)** Distribution of CD206$^{+}$ macrophages (green) and CD206$^{-}$ macrophages (grey) between the two groups.

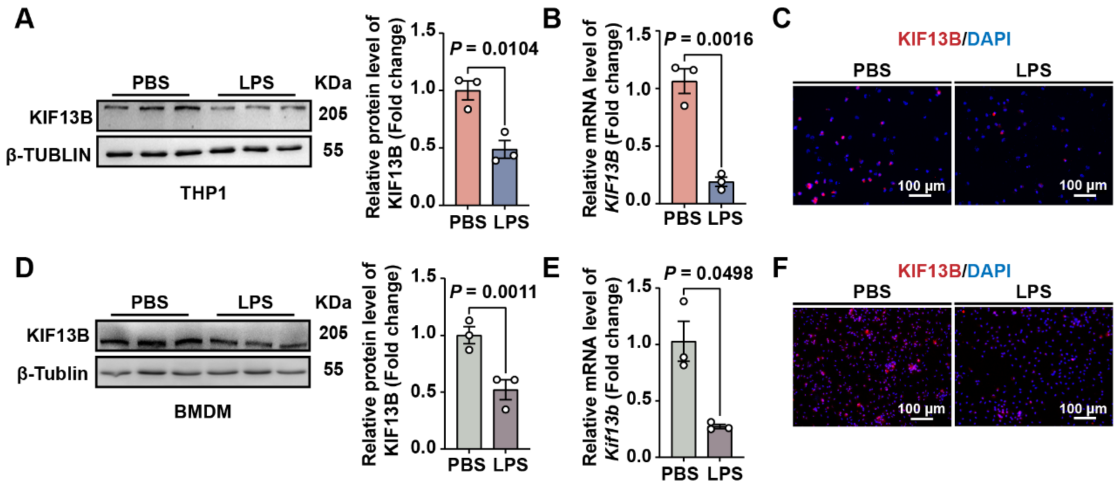


**Fig S2. KIF13B levels are reduced in LPS-treated macrophages.**

**(A-C)** THP1-derived macrophages were stimulated with PBS or 10 ng/ml LPS for 24 h (n = 3/group). Western blot **(A)** and qPCR **(B)** analysis of KIF13B mRNA and protein levels, respectively, and immunofluorescence staining images **(C)** of KIF13B (red) in each group. **(D-F)** Primary mouse BMDMs extracted from WT and *Kif13b*$^{-/-}$ mice were stimulated with PBS or 10 ng/ml LPS for 24 h. Western blot **(D)** and qPCR **(E)** analysis of KIF13B mRNA and protein levels, respectively, and immunofluorescence staining images **(F)** of KIF13B (red) in each group. Data were presented as mean ± SEM. Data **A-C** were analyzed by unpaired Student's *t*-test. Data **D** was analyzed by Welch t-test.

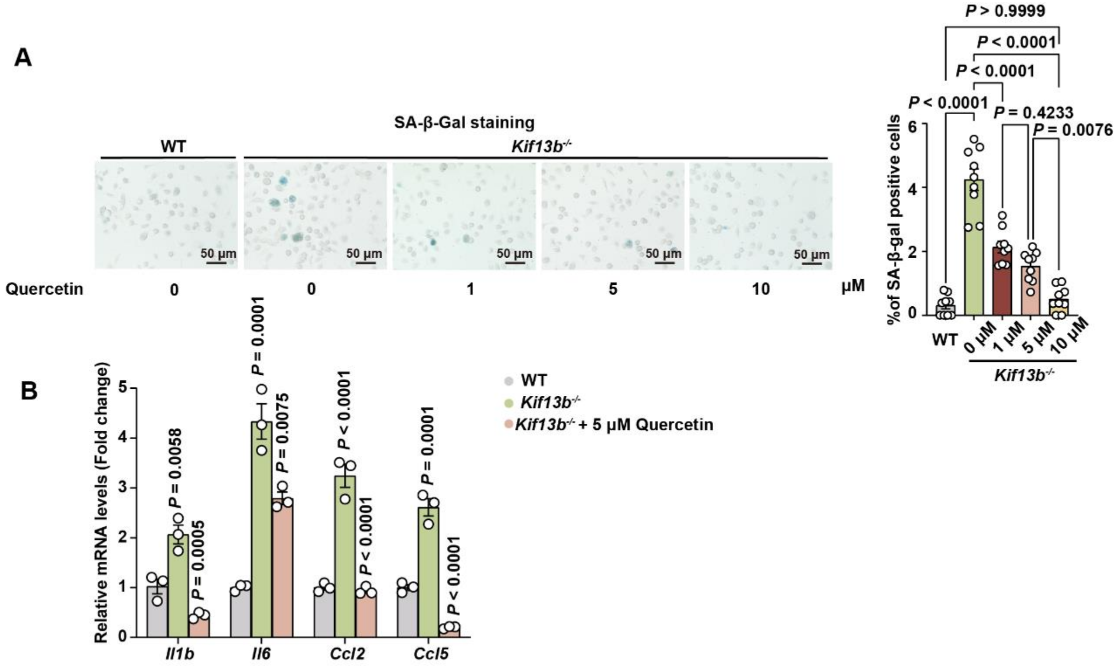


**Fig S3. Senolytic therapy inhibits macrophage pro-inflammation caused by *Kif13b* deficiency.**

**(A)** Representative images of SA-β-gal staining of primary mouse BMDMs isolated from 8-week-old male WT and *Kif13b*$^{-/-}$ mice that were treated with DMSO or quercetin at the indicated concentrations for 24 h (Left); Quantitative analysis of the percentage of SA-β-gal positive cells (n = 9/group) (Right). **(B)** qPCR analysis of the mRNA expression of pro-inflammatory genes in WT and *Kif13b*$^{-/-}$ macrophages with or without a treatment of 5 μM quercetin for 24 h (n = 3/group). Data were presented as mean ± SEM and analyzed by one-way ANOVA with Bonferroni tests.

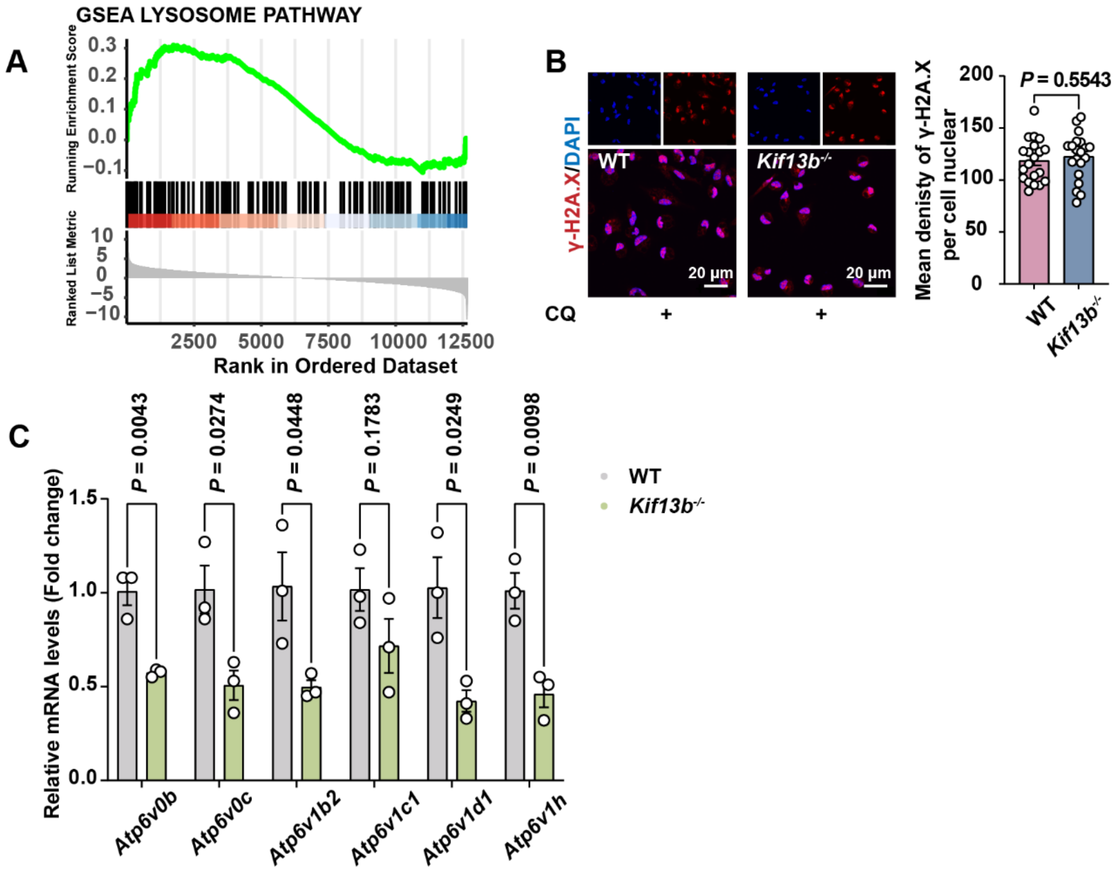


**Fig S4. Loss of KIF13B promotes lysosomal dysfunction.**

**(A)** GSEA analysis of lysosome pathway in the transcriptome data of WT and *Kif13b*$^{-/-}$ primary mouse BMDMs. Pathways with NES greater than 1.5 and P value less than 0.05 were identified as a significant enrichment. **(B)** Representative images of immunofluorescence staining for γ-H2A.X (red) and DAPI (blue) in WT and *Kif13b*$^{-/-}$ primary mouse BMDMs pretreated with CQ for 1 h (n = 3 biologically independent experiments/group). **(C)** qPCR analysis of the mRNA expression of genes associated with V-ATPases complex in WT and *Kif13b*$^{-/-}$ mouse primary macrophages (n = 3/group). Data were presented as mean ± SEM and analyzed by unpaired Student's *t*-test.

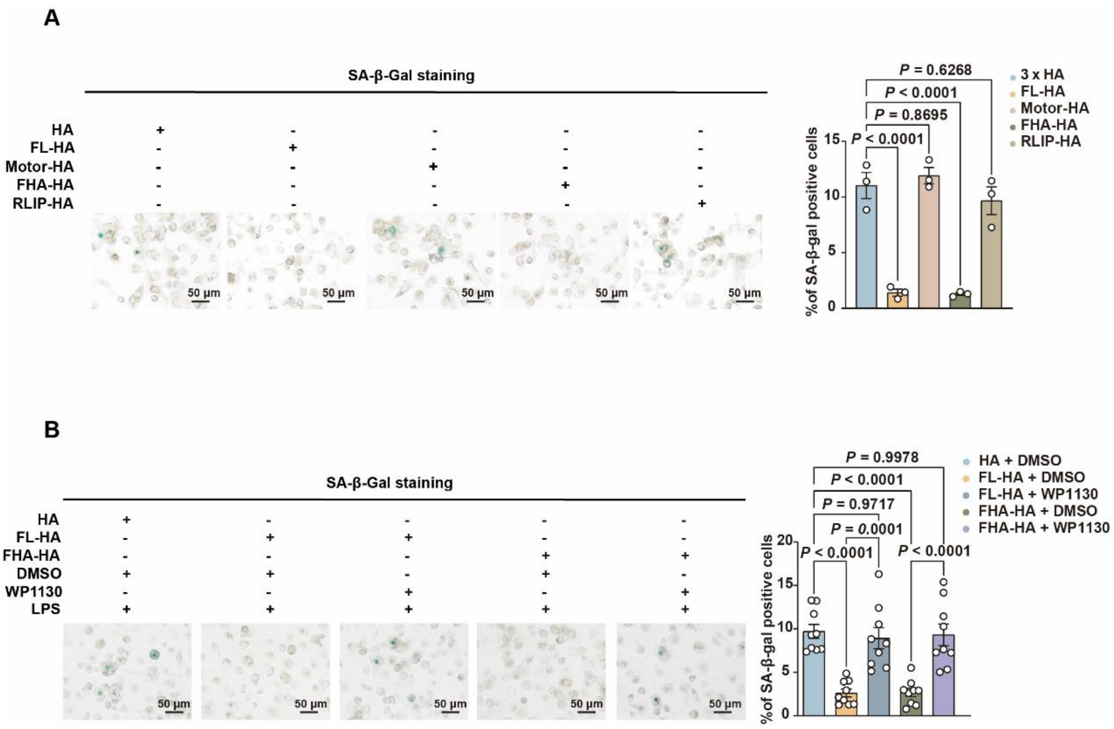


**Fig S5. The FHA domain of KIF13B attenuates macrophage senescence in a USP9X-dependent manner.**

**(A)** Representative images of SA-β-gal staining of THP1-derived macrophages transfected with full-length *KIF13B* plasmid (FL-HA), KIF13B-Motor plasmid (Motor-HA), KIF13B-FHA plasmid (FHA-HA), KIF13B-RLIP plasmid (RLIP-HA), or HA-tagged empty vector control then treated with 10ng/ml LPS for 24 h (Left); Quantitative analysis of the percentage of SA-β-gal positive cells (n = 3/group) (Right). **(B)** Representative images of SA-β-gal staining of THP1-derived macrophages transfected with full-length *KIF13B* plasmid (FL-HA), KIF13B-FHA plasmid (FHA-HA) or HA-tagged empty vector control then treated with 10ng/ml LPS together with DMSO or WP1130 for 24 h (Left); Quantitative analysis of the percentage of SA-β-gal positive cells (n = 9/group) (Right).

**Table S1. Plasma lipids in PPE-induced AAA mice.**

| Genotypes | TC (mg/dL) | TG (mg/dL) |
|---|---|---|
| WT | 119.5 ± 2.113 | 68.45 ± 4.068 |
| *Kif13b*$^{-/-}$ | 143.6 ± 2.315(p<0.0001) | 70.17 ± 2.569 |
| *Kif13b*$^{f/f}$ | 116.7 ± 3.342 | 77.97 ± 1.865 |
| *Sm22Kif13b*$^{f/f}$ | 112.3 ± 3.952 | 69.89 ± 3.446 |
| *Kif13b*$^{f/f}$ | 107.0 ± 1.718 | 79.87 ± 2.329 |
| *Lyz2Kif13b*$^{f/f}$ | 108.0 ± 2.617 | 74.37 ± 1.777 |

Data were presented as mean ± SEM. Data were analyzed by unpaired Student's *t*-test. n = 8/group.

**Table S2. Blood pressure in ANGII/PCSK9-inducedAAAmice.**

| Group | Basline | | Modelling | |
|---|---|---|---|---|
| | WT | *Kif13b*$^{-/-}$ | WT | *Kif13b*$^{-/-}$ |
| SBP (mmHg) | 91.99 ± 2.352 | 98.32 ± 1.728 | 145.9 ± 5.016 (p < 0.0001) | 146.9 ± 4.851 p > 0.9999 |
| DBP (mmHg) | 70.35 ± 2.282 | 73.64 ± 0.7007 | 107.9 ± 4.608 (p < 0.0001) | 104.4 ± 5.268 p > 0.9999 |

Data were presented as mean ± SEM. Data SBP were analyzed by two-way ANOVA with Bonferroni tests. n = 6/group.

## Table S3. The list of siRNA sequence for Human *KIF13B* gene detection

| Gene name | Sequences Sense (5' -3') | Anti-sense (5' -3') |
|---|---|---|
| si*KIF13B*-1 | CCUCCAUGAAGAACGAGAAUATT | UAUUCUCGUUCUUCAUGGAGGTT |
| si*KIF13B*-2 | CCCAGUAAUACGAUCAUACUUTT | AAGUAUGAUCGUAUUACUGGGTT |
| si*KIF13B*-3 | GCCUUGAAGAUCUGCGACAAATT | UUUGUCGCAGAUCUUCAAGGCTT |

**Table S4. The list of mouse primer sequences**

| Gene name | Forward primer | Reverse primer |
|---|---|---|
| *Kif13b* | AGGAGGGCAGCAACATCAACAA | TGTCCTTGAGCAGCCATGTGAG |
| *Il1b* | TGGACCTTCCAGGATGAGGACA | GTTCATCTCGGAGCCTGTAGTG |
| *Il6* | GACCTGTCTATACCACTTCAC | GTGCATCATCGTTGTTCATAC |
| *Ccl2* | CCACTCACCTGCTGCTACTCAT | CACTGTCACACTGGTCACTCCT |
| *Ccl5* | CCTCACCATCATCCTCACTG | CACACTTGGCGGTTCCTTC |
| *p16$^{INK4a}$* | GCTGGGTGCTCTTTGTGTTC | TCTGCTCCCTCCCGTGATT |
| *p19$^{ARF}$* | TCACCTCGCTTGTCACAGT | GCACCGTAGTTGAGCAGAAG |
| *p21* | GTTCCTTGCCACTTCTTACCT | GTTGAGTCCTAACTGCCATCC |
| *TFEB* | CGCCTGGAGATGACTAACAAG | GGTGATGGAACGGAGACTGT |
| *Atp6v1h* | GCCATACTTCCTGCCAATGTT | GTTCACTCCATCTGCTTCTACC |
| *Atp6v1a* | ACTTCTGGTGTCTCTGTTGGA | GTCACGCTTCCTCTGTTACG |
| *Atp6v1c1* | CAGCGACCACCAAGAACAATA | TCATCCGACAAGCCAACCA |
| *Atp6v1b2* | CAGCCTCGTCTCACCTACAA | TCTTCAGCCAGCACCACAG |
| *Atp6v1d1* | CGTGGACAATGGCTACTTGG | TGTGGCGGAACTCTACTACC |
| *Atp6v0b* | TCAGACCTCCAGAGTGAAGATG | AGAGTCAACAGGCAGCAAGA |
| *Gapdh* | CCAAGGTCATCCATGACAACTT | AGGGGCCATCCACAGTCTT |

**Table S5. The list of human primer sequences**

| Gene name | Forward primer | Reverse primer |
|---|---|---|
| *IL1B* | GGACAGGATATGGAGCAACAAG | TTCAACACGCAGGACAGGTA |
| *IL6* | CTTCGGTCCAGTTGCCTTCT | GGCTTGTTCCTCACTACTCTCA |
| *CCL2* | AAAGTCTCTGCCGCCCTTC | CTTGCTGCTGGTGATTCTTCTA |
| *CCL5* | GCTGTCATCCTCATTGCTACTG | CACACACTTGGCGGTTCTTT |
| *GAPDH* | CAAGGCTGTGGGCAAGGTCATC | GTGGGTGTCGCTGTTGAAGTCA |
